# Automated Implementation of the Edinburgh Visual Gait Score (EVGS) Using OpenPose and Handheld Smartphone Video

**DOI:** 10.3390/s23104839

**Published:** 2023-05-17

**Authors:** Shri Harini Ramesh, Edward D. Lemaire, Albert Tu, Kevin Cheung, Natalie Baddour

**Affiliations:** 1 Department of Mechanical Engineering, University of Ottawa, Ottawa, ON K1N 6N5, Canada; nbaddour@uottawa.ca; 2 The Ottawa Hospital Research Institute, Ottawa, ON K1H 8M2, Canada; elemaire@ohri.ca; 3 Faculty of Medicine, University of Ottawa, Ottawa, ON K1H 8M5, Canada; 4 Department of Surgery, Division of Neurosurgery, Children’s Hospital of Eastern Ontario, Ottawa, ON K1H 8L1, Canada; atu@cheo.on.ca; 5 Department of Surgery, Division of Plastic Surgery, Children’s Hospital of Eastern Ontario, Ottawa, ON K1H 8L1, Canada; kcheung@cheo.on.ca

**Keywords:** gait analysis, Edinburgh Visual Gait Score, computer vision, motion analysis, pose estimation, smartphone video, remote gait analysis

## Abstract

Recent advancements in computing and artificial intelligence (AI) make it possible to quantitatively evaluate human movement using digital video, thereby opening the possibility of more accessible gait analysis. The Edinburgh Visual Gait Score (EVGS) is an effective tool for observational gait analysis, but human scoring of videos can take over 20 min and requires experienced observers. This research developed an algorithmic implementation of the EVGS from handheld smartphone video to enable automatic scoring. Participant walking was video recorded at 60 Hz using a smartphone, and body keypoints were identified using the OpenPose BODY25 pose estimation model. An algorithm was developed to identify foot events and strides, and EVGS parameters were determined at relevant gait events. Stride detection was accurate within two to five frames. The level of agreement between the algorithmic and human reviewer EVGS results was strong for 14 of 17 parameters, and the algorithmic EVGS results were highly correlated (r > 0.80, “r” represents the Pearson correlation coefficient) to the ground truth values for 8 of the 17 parameters. This approach could make gait analysis more accessible and cost-effective, particularly in areas without gait assessment expertise. These findings pave the way for future studies to explore the use of smartphone video and AI algorithms in remote gait analysis.

## 1. Introduction

Gait analysis is used to evaluate functional status and neurological health, particularly for those with mobility issues [1,2]. Instrumented gait analysis (IGA) is the gold standard that uses three-dimensional body pose data to diagnose gait abnormalities [3,4,5]. However, IGA is resource-intensive and not available in most clinical settings [4]. Observational gait analysis or visual gait analysis (VGA) is an alternative that provides a simple and easy-to-use procedure for clinicians to observe and characterize a patient’s gait. In VGA, recorded video is evaluated using a variety of scales that concentrate on specific joints, planes, and events in the gait cycle. Despite its subjective nature, VGA is widely used to evaluate gait issues in children and adults, and several computer-based image analysis methods have been designed to support clinicians. However, VGA may result in low sensitivity, specificity, validity, and reliability when compared to IGA [6].

One of the oldest VGA scoring scales is the Physician Rating Scale (PRS), which was specifically developed for children with cerebral palsy (CP) [7]. Its development, reliability, and validity were not well documented when the scale was first published in 1993. Since then, several other VGA scales have been proposed for different patient populations or to improve the validity of existing scales [8,9,10,11,12,13,14,15,16,17,18]. A review that analyzed five VGA gait measures found that the PRS was insufficient to capture the entire gait pattern accurately [19]. The Salford Gait Tool was highly reliable and concurrently valid, but only assessed sagittal plane gait deviation and required additional validation [19]. The Edinburgh Visual Gait Score (EVGS) was the most effective scale for evaluating gait patterns of children with CP because it included parameters for the trunk, pelvis, hip, knee, and ankle in both the stance and swing phases and data on gait in all three planes [20].

The EVGS has emerged as a reliable tool for grading patient walking videos, particularly for children with cerebral palsy [19]. However, existing methods still require human assistance and can be time-consuming to implement, with the time to grade a video for one patient taking up to 24.7 min [21]. While previous research explored automatic gait analysis, such as the research by Yoo et al. [22], their approach did not utilize handheld videos or scoring scales. Another study by Myriam et al. [23] developed a software package for automated gait analysis based on the Rancho Observational Gait Analysis approach, identifying gait deviations and their causes. However, their software was not tested, and some issues needed to be resolved, including automatic filling of the tables and synchronization with Visual3D analysis software.

To address these limitations, automated tools could be developed for the EVGS analysis of video clips with minimal human interaction. In this context, smartphones can play a valuable role in acquisition and gait monitoring, providing high-quality video recordings of human movement that can be analyzed using artificial intelligence (AI) algorithms. This would enable gait analysis from any location, including rural clinics, and improve access to care for patients with mobility issues.

Deep learning methods have shown the most promise for accurate pose estimation, and various techniques are available, including top-down and bottom-up approaches [24,25,26,27]. OpenPose, HyperPose, and BlazePose are among the most widely used markerless techniques for pose estimation. OpenPose is a reliable markerless motion analysis system for pose estimation, particularly for gait analysis [26]. Research comparing OpenPose and HyperPose performance showed that OpenPose BODY25 consistently classified body keypoints more accurately than HyperPose [28], while other studies found that OpenPose performed better than BlazePose when predicting human pose estimation coordinates [29]. Although OpenPose requires more computational resources compared to other models, it offers more accurate keypoint detection, which is crucial for gait analysis. As shown in Table 1, when compared to other widely used pose estimation methods, OpenPose has the necessary inputs to compute the EVGS, making it a suitable option for automated gait analysis using smartphone video recordings.

The goal of this research was to create a viable system that can automatically detect foot events and strides and then calculate the EVGS from handheld smartphone video. This work aimed to determine whether automatic EVGS analysis can be performed using OpenPose keypoints alone. An automated system must be time-efficient to reduce the workload for clinicians. A viable system will offer a novel, automated implementation of visual gait analysis using smartphone video, making this level of gait analysis more accessible and convenient for patients and more time efficient for clinicians.

The proposed system makes these main contributions:

- Provides viable methods that use handheld smartphone video, thereby making this approach applicable to the remote video capture of patient movements by clinicians or parents/caregivers;
- Creates an automatic system using OpenPose keypoints to detect foot events and strides and automatically calculate the EVGS. This potentially reduces the workload for clinicians because the EVGS is calculated quickly and automatically without the need for manual calculations or human intervention;
- This approach saves substantial time and resources, eliminating the need for specialized equipment or extensive preparation for gait analysis.

The research presents a novel and time-efficient method for calculating the EVGS results using smartphone video data, advancing the understanding of automatic gait analysis methods using the EVGS and providing a basis for future research in this area. Overall, this research provides a promising avenue for the integration of automatic EVGS analysis and scoring using smartphones, paving the way for remote visual gait analysis technology.

## 2. Materials and Methods

To achieve the automated EVGS analysis objective, the proposed system must be able to identify the appropriate video frames for analysis and then apply a series of rules to generate scores for each parameter. This involves two main tasks: creating a method for identifying the required gait events in a walking video and creating an algorithm for scoring the EVGS parameters. The proposed methodology sequentially implements pose estimation, view detection, direction of motion detection, gait event identification, stride allocation, and finally, algorithmic calculation of the EVGS.

A preliminary set of videos was needed to develop the algorithm. To obtain these videos, an able-bodied individual walking in both sagittal and coronal views was recorded at 60 Hz using a handheld smartphone camera.

### 2.1. Algorithm Development

#### 2.1.1. Pose Estimation

**Figure 1:**
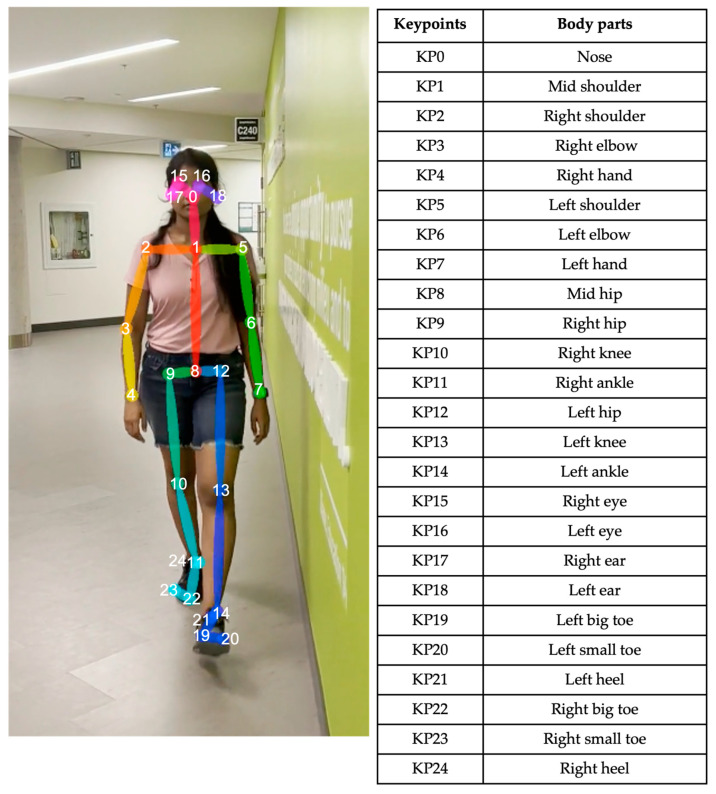
OpenPose output demonstrating human pose estimation through keypoint detection.

For each video frame, the OpenPose BODY25 model provided joint coordinates as keypoints (Figure 1). Coordinate trajectories can be noisy and sometimes contain outliers; therefore, keypoint data were filtered using a zero-phase, dual-pass, second-order Butterworth filter with a 12 Hz cut-off frequency. A 2D (two-dimensional) keypoint processing strategy was adapted from earlier work for a markerless AI motion analysis method for hallways [32], where keypoints with scores below a 10% threshold were removed, and then cubic spline interpolation was used to fill gaps of five frames (0.083 s) or fewer.

#### 2.1.2. Gait Event and Stride Detection

The process involved determining if the video is in the sagittal or coronal plane; determining whether the motion is left to right, right to left, anterior to posterior, or posterior to anterior; detecting the four distinct gait events; and assigning values to a particular stride. The required events are foot strike, foot off, mid-midstance (midpoint of midstance) and mid-midswing (midpoint of midswing).

##### Detection of Sagittal/Coronal Views

Trunk length fluctuates very little when walking in the sagittal plane, and trunk length stays within a certain threshold. The algorithm considers a video to be sagittal when the absolute difference in trunk length (distance between the midshoulder (KP1) and midhip (KP8) keypoints) between the first and last frames is less than a threshold (99 pixels). To identify the threshold, 37 videos of able-bodied people walking in both coronal and sagittal views were analyzed. A difference in trunk length of less than 99 pixels produced the best results, where the video resolution was 1920 × 1080. Therefore, 99 pixels was set as the threshold for classifying sagittal plane walking.

In the front (coronal) view, trunk length is maximum when the person is close to the camera and decreases from maximum when the person walks away from the camera.

##### Direction of Motion Detection

Following the coronal or sagittal identification of the video, the next step determines frontal or rear in coronal view and left to right or right to left in sagittal view.

An algorithm was employed that replicated the approach for locating the sagittal/coronal views by monitoring trunk length and comparing the difference between the first and final frames. If the difference is negative, the participant is facing forward, and if the difference is positive, the person is moving away from the camera.

In the sagittal view, the algorithm tracks the nose (KP0) location on the x-axis and calculates the difference between nose positions in the first and final frames. X-coordinates are maximal at the right border and minimal at the left border. Using this concept, if the difference is negative, the person is walking from left to right. If the difference is positive, the person is walking from right to left. This method produced consistent results.

##### Gait Event Identification

**Figure 2:**
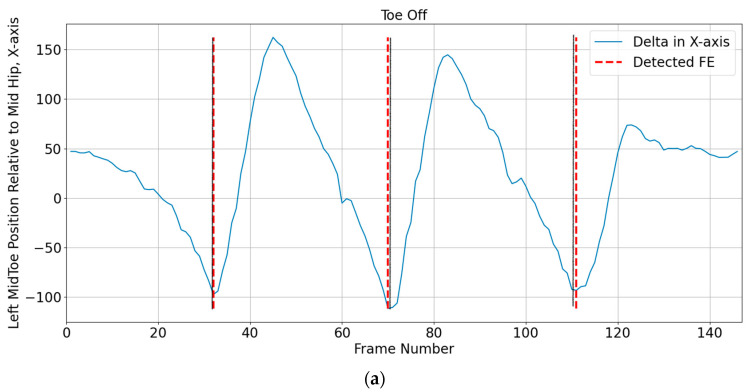

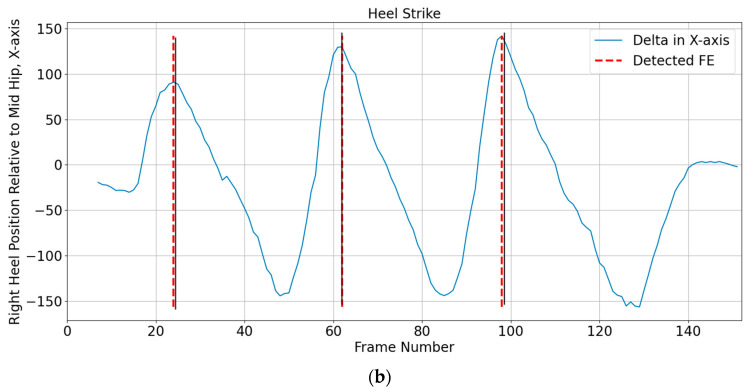
Identification of heel strike and toe off for right leg (**a**) midtoe position, (**b**) heel position. Solid black line represents actual foot event frame, and dashed red line is estimated foot event. FE = foot event.

Foot strike and foot-off events were determined using the Zeni et al. [33] method. According to this approach, heel strike is when the sacral marker’s forward distance from the heel marker is at its maximum, and toe off is when the toe marker is furthest posterior from the sacral marker. Since sacrum keypoints are not provided in OpenPose, the midhip keypoint (KP8) was used to replace the sacral marker location. Figure 2 provides an example of identified foot strikes and foot off using the heel positions (KP24 and KP21) and midhip positions (KP8).

**Figure 3:**
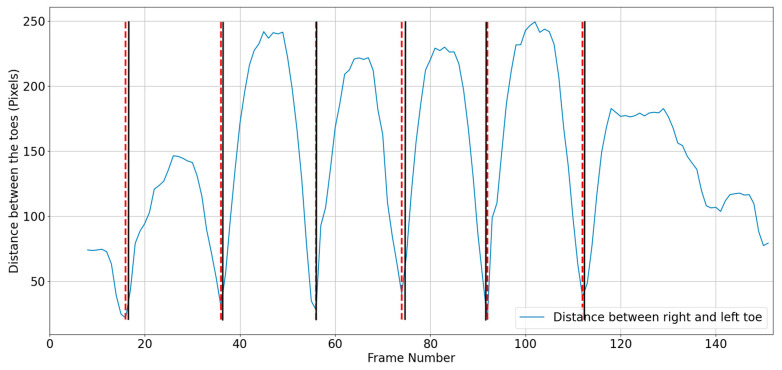
Mid-midstance detection using toe distance. The dashed red line shows the algorithmically calculated mid-midstance, whereas the solid black line shows actual frame number of the mid-midstance.

The distance between the toes (KP19 and KP22) is used to determine mid-midstance and mid-midswing. The legs are closer together in mid-midstance posture; hence, the space between the two toes will be less. Assuming that the right big toe (KP22) is ($x_{1}$, $y_{1}$) and the left big toe (KP19) is ($x_{2}$, $y_{2}$), the distance between toes (d) was calculated using Equation (1), and mid-midstance was at the minimum distance (Figure 3):

(1) $$d=\sqrt{{\left(x_{2}-x_{1}\right)}^{2}+{\left(y_{2}-y_{1}\right)}^{2}}$$

**Figure 4:**
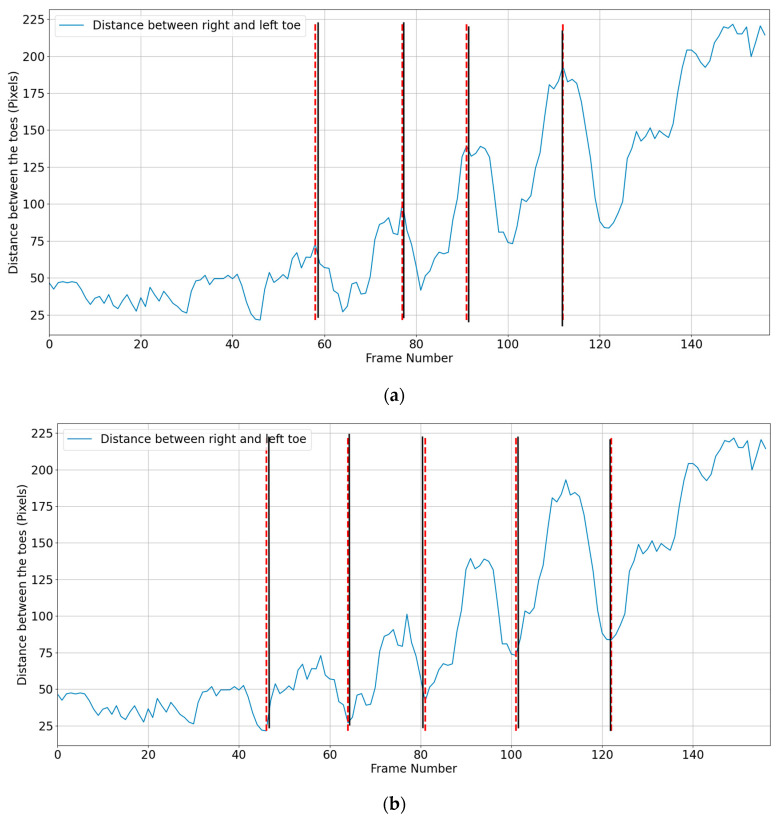
Distance between the toes during a walking trial: (**a**) heel strike and (**b**) mid-midstance. The red dashed line is algorithm’s estimated mid-midstance and heel strike. Solid black lines are ground truth for mid-midstance and heel strike.

For a coronal view, the beginning and end of the stride, and mid-midstance must be located. The same procedure was used as in the sagittal view, measuring the distance between the toes (Figure 4).

##### Allocating Foot Events to Specific Strides

**Figure 5:**
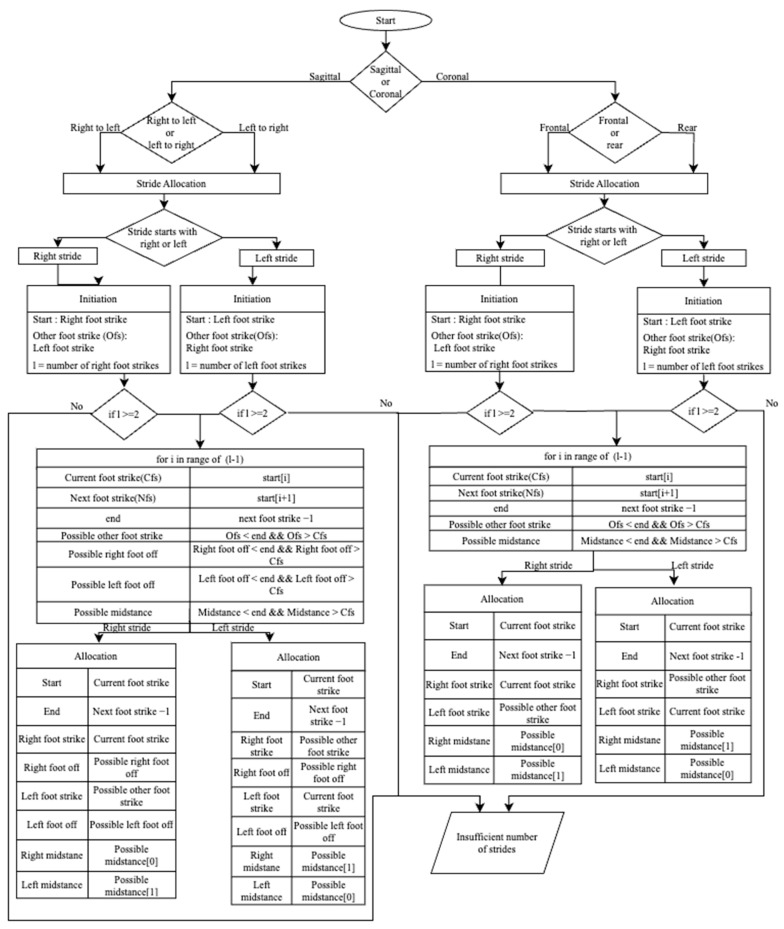
Flowchart of gait event identification.

Figure 5 provides a flowchart for identifying strides. The method involves determining if the stride begins on the left or the right; acquiring at least a stride to determine if the number of right/left foot strikes equals or exceeds 2; and assigning events to particular strides by searching for foot strikes, foot offs, or mid-midstance that could happen between the start and finish of the stride.

#### 2.1.3. Algorithmic Implementation of EVGS

The Edinburgh Visual Gait Score (EVGS) is a clinical tool used to assess gait deviations in patients with various neurological and musculoskeletal conditions. EVGS consists of 17 parameters for each lower extremity, totaling 34 parameters. In practice, a person’s gait in sagittal and coronal views are video recorded while walking, using a handheld camera or a camera on a tripod. In typical clinical practice, the video is scored by a human according to the EVGS guidelines. The person scoring the video can use video editing software to pause the recorded video at specific gait events for analysis. Software can also be used to determine joint angles and other EVGS parameters. A 3-point ordinal scale is used to score each parameter: 0 = normal (within +/− 1.5 standard deviations of the mean), 1 = moderate deviations (between 1.5 and 4.5 standard deviations of the mean), and 2 = significant/severe deviations (more than 4.5 SD of mean). A lower overall score indicates less gait deviation [20]. In this research, to enhance understanding and maintain consistency, the 17 EVGS parameters were grouped based on foot events and gait cycle phases. Table 2 below lists the parameters and how they are organized according to these events.

**Figure 6:**
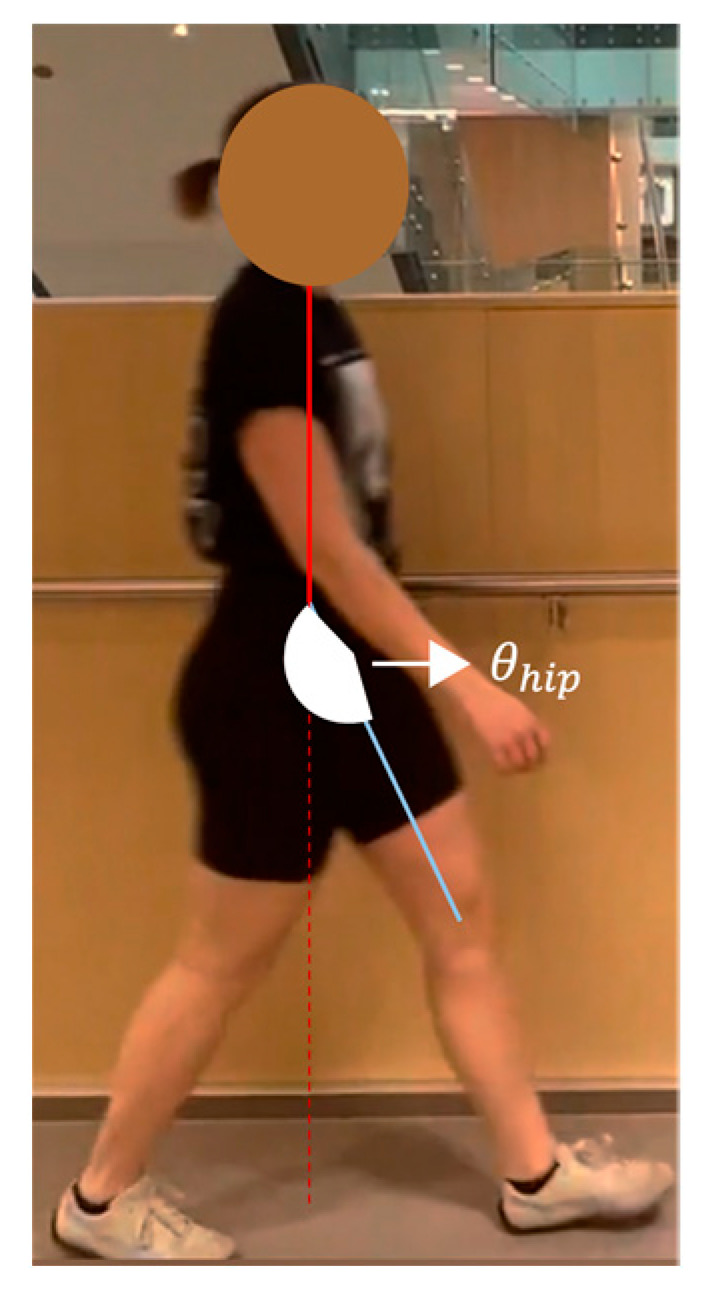
Geometric representation of the method used to calculate hip angle.

The majority of EVGS parameters need angle calculations, including hip, knee, and ankle. Hip angle in the sagittal plane was computed using the angle between the axis perpendicular to the trunk axis and the thigh axis (Figure 6), Since OpenPose does not provide keypoints for a pelvis segment. The trunk axis was the axis between the midshoulder (KP1) and midhip (KP8) keypoints. The line connecting the hip (KP9 and KP12) and knee (KP10 and KP13) keypoints was the thigh axis. Note that this is different from how people can visualize hip angle, which is usually the thigh angle related to pelvis orientation. Equation (2) was used to calculate the slope

(2) $$m=\frac{y_{2}-y_{1}}{x_{2}-x_{1}}$$

**Figure 7:**
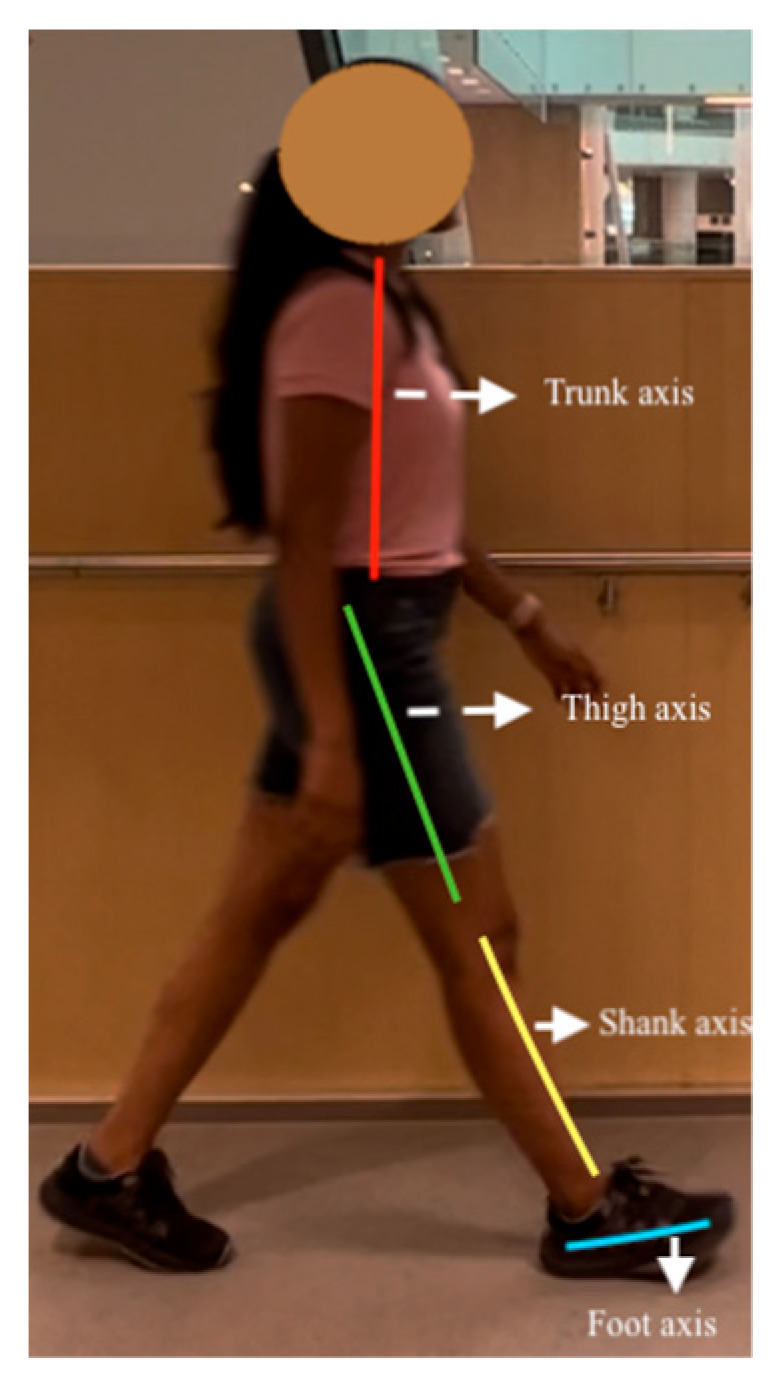
Axes for EVGS computation.

For the trunk axis, ($x_{1}$, $y_{1}$) are the midshoulder (KP1) coordinates, and ($x_{2}$, $y_{2}$) are the midhip (KP8) coordinates. Using these coordinates and Equation (3), the trunk axis angular coefficient was determined. The axes used to compute joint angles are shown in Figure 7. The axis perpendicular to the trunk axis thus had the following slope:

(3) $$m_{1}=\frac{1}{m}$$

A similar procedure was followed for the thigh axis, where $m_{2}$ was calculated from ($x_{3}$,$y_{4}$), which corresponds to the hip’s coordinates (KP9 and KP12), and ($x_{4}$, $y_{4}$) represents the knee keypoints (KP10 and KP13):

(4) $$m_{2}=\frac{y_{4}-y_{3}}{x_{4}-x_{3}}$$

Therefore, the angle (in degrees) is given by

(5) $$\theta_{hip}=90-arctan\left\|\frac{m_{2}-m_{1}}{1+\left(m_{2}\times m_{1}\right)}\right\|\times \frac{360}{2\pi }$$

When the knee is in front of the body, the angle value is positive (flexion). This method was used to calculate peak hip flexion in swing (#13) and peak hip extension in stance (#12).

**Figure 8:**
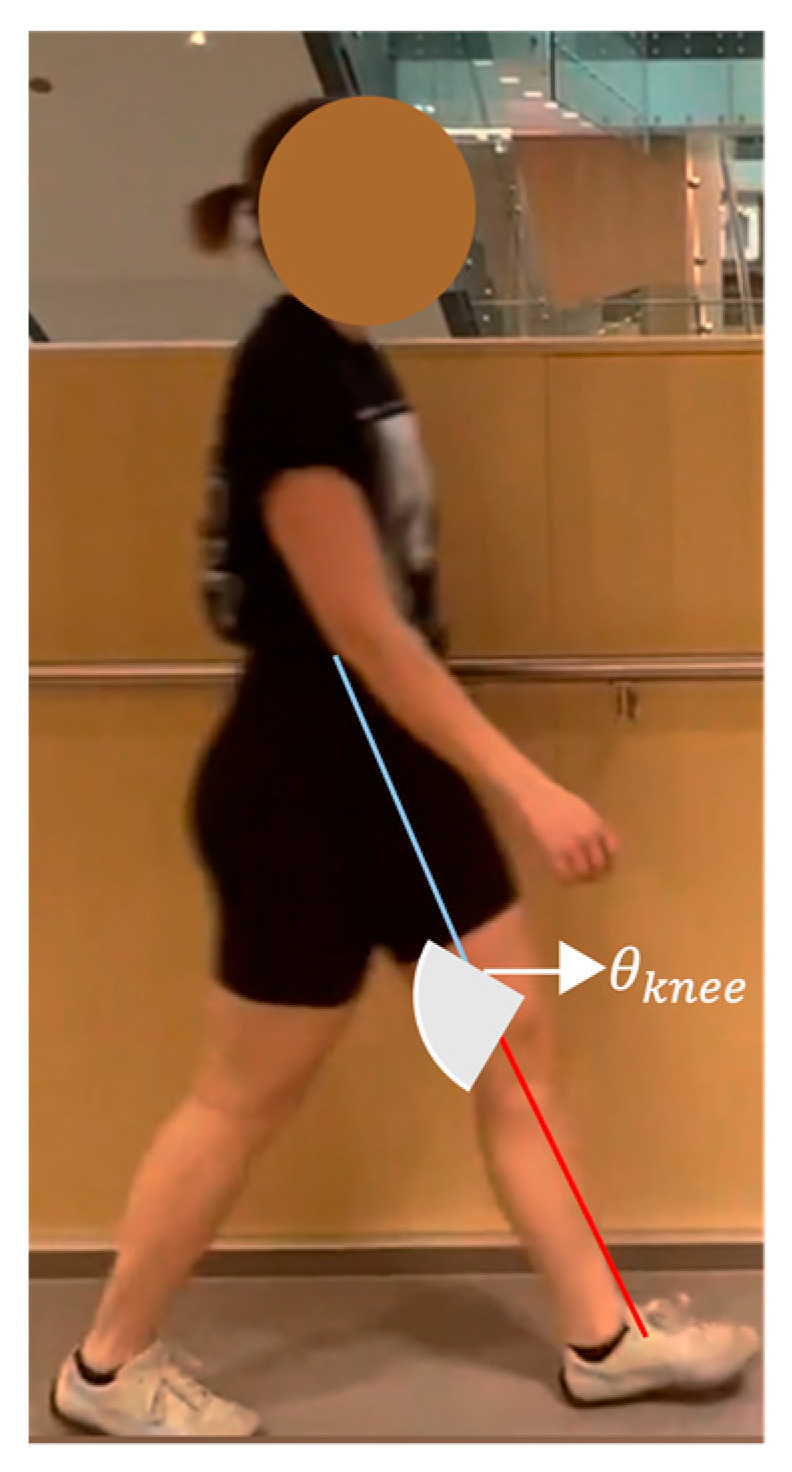
Geometric representation of the method used to calculate knee angle.

Knee extension in terminal swing is determined using the knee angle, the angle between the thigh and shank sagittal axes (Figure 8). When the knee is flexed, the angle is positive. Equation (4) determines the slope of the thigh segment, and Equation (6) is for the tibia segment slope. 

For the thigh, hip coordinates (KP9 and KP12) were ($x_{3}$, $y_{3}$), and knee coordinates (KP10 and KP13) were ($x_{4}$, $y_{4}$), and Equation (4) was used to compute the angular coefficient (slope) of thigh axis $m_{2}$. The ankle coordinates (KP11 and KP14) were ($x_{5}$, $y_{5}$). The slope, $m_{3}$ (shank axis), was calculated using Equation (6):

(6) $$m_{3}=\frac{y_{5}-y_{4}}{x_{5}-x_{4}}$$

Knee angle was then calculated using

(7) $$\theta_{knee}=arctan\left\|\frac{m_{3}-m_{2}}{1+\left(m_{3}\times m_{2}\right)}\right\|\times \frac{360}{2\pi }$$

This method was used to calculate knee extension in terminal swing (#10), peak knee extension in stance (#9), and peak knee flexion in swing (#11).

**Figure 9:**
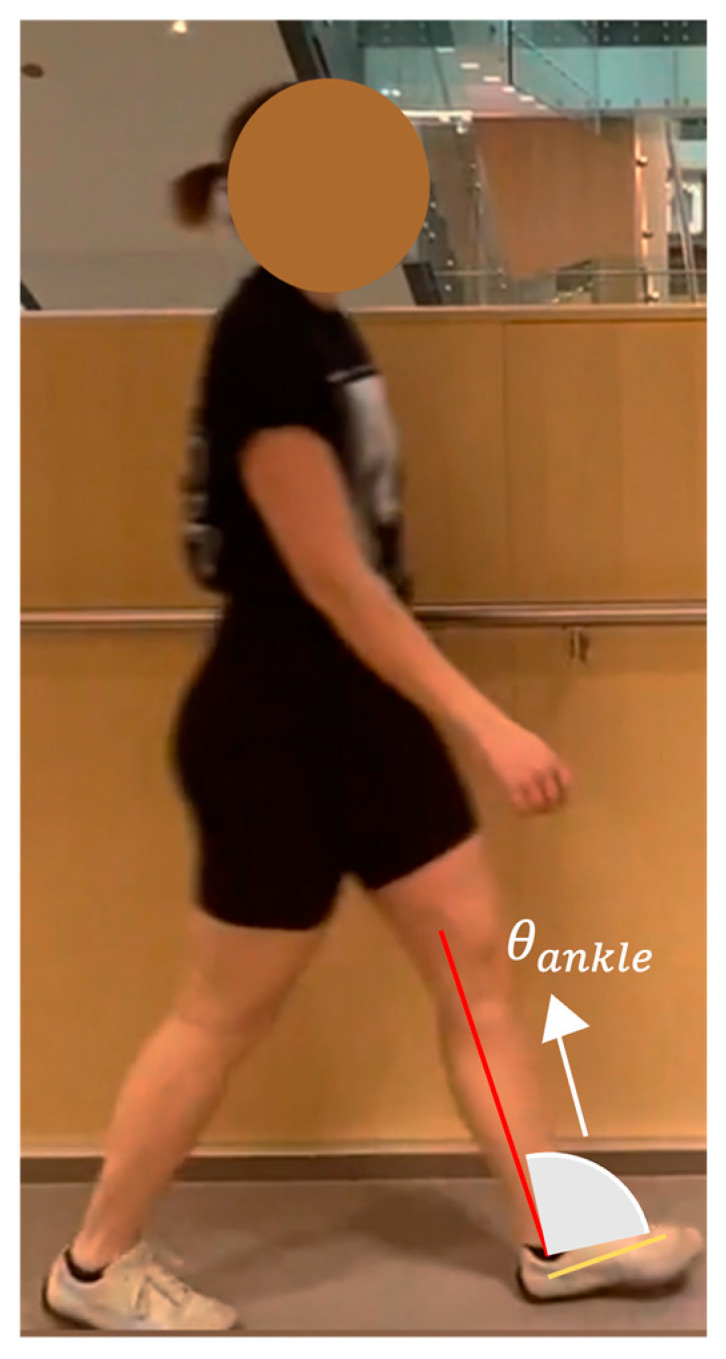
Geometric representation of the method used to calculate ankle angle.

The dorsiflexion/plantarflexion angle was calculated using the foot and shank sagittal axes (Figure 9). 

Dorsiflexion angles are positive. Equations (6) and (8) are used for the tibia and foot segments. The knee coordinates (KP10 and KP13) are ($x_{4}$, $y_{4}$), and ankle coordinates (KP11 and KP14) are ($x_{5}$, $y_{5}$). Using Equation (6), the angular coefficient is $m_{3}$ (shank axis). The heel coordinates (KP21 and KP24) are ($x_{6}$, $y_{6}$), and the mean positions of the big (KP19 and KP22) and small toes (KP20 and KP23) are ($x_{7}$, $y_{7}$). Equation (8) determines the slope $m_{4}$(foot axis)

(8) $$m_{4}=\frac{y_{7}-y_{6}}{x_{7}-x_{6}}$$

The ankle angle was calculated using Equation (9):

(9) $$\theta_{ankle}=arctan\left\|\frac{m_{4}-m_{3}}{1+\left(m_{4}\times m_{3}\right)}\right\|\times \frac{360}{2\pi }$$

##### Initial Contact (#1)

To determine whether the heel, toe, or flat foot makes initial contact with the ground, a line (foot axis) between the heel (KP21 and KP24) and midtoe (midpoint of the big (KP19 and KP22) and small toe (KP20 and KP23)) keypoints were used. The angle between the foot axis and the image coordinate system x-axis in sagittal plane was measured. The development dataset was used to calculate angle thresholds for scoring. The thresholds were determined by evaluating sagittal videos showing any of the three conditions (heel contact, toe contact, flat foot contact). After analysis, the flat foot contact range was set to between 0 and 20°, Since 95% of the development dataset showed heel contact at more than 20° and toe contact at less than 0°.

##### Peak Sagittal Trunk Position (#16)

Similar to the foot position approach, a line from the midshoulder (KP1) to the midhip keypoint (KP8) was used for trunk angle. The angle between this trunk line and the x-axis of the image coordinate system in the sagittal plane was calculated. Then, in accordance with the EVGS handbook [20], normal (vertical to 5° forward or backward), abnormal (greater than 5° backward or between 6° and 15° forward), and highly abnormal (more than 15° forward) conditions were classified.

##### Pelvic Rotation in Midstance (#15) and Maximum Pelvic Obliquity in Stance (#14)

OpenPose only provides hip keypoints, but the pelvis requires at least 3 keypoints to define segment orientation. As a surrogate measure, pelvic rotation angle was determined from a line connecting the right (KP9) and left hip (KP12) joints and the image coordinate system’s sagittal plane y-axis.

##### Heel Lift (#2)

Heel lift has five EVGS criteria: normal, early, delayed, no heel, and no forefoot contact. Using the same methodology for detecting foot position at initial contact, no forefoot touch and no heel contact were detected at midstance. “No forefoot contact” refers to when the forefoot (the front part of the foot, including the toes) does not contact the ground during midstance. “No heel contact” refers to when the heel does not contact the ground during midstance.

Three foot events (stance foot heel lift, other foot level with the stance foot, opposite foot contact with the ground) were recorded if the foot was in a flat posture during midstance. A normal EVGS result occurs if heel lift occurs on the stance leg between the other foot being at the same level as the stance foot and the other foot making contact with the ground. Heel lift is early if the stance leg heel lift occurs before the opposing foot levels with the stance foot. Heel lift is delayed if lifting occurs after the opposing foot reaches the ground.

##### Foot Clearance in Swing (#6)

Foot clearance has four criteria: full clearance, reduced clearance, no clearance, and high steps. Toe, heel, and ankle keypoints were considered for this parameter. Full clearance occurs when the big toe and heel involved in foot clearance cross the big toe and heel of the opposing leg, respectively. Reduced clearance occurs when the heel crosses the opposing leg’s heel, but the toe remains below or on the same level as the opposite leg’s toe. No clearance is when both big toe and heel are below the opposite big toe and heel, respectively. A high step occurs if the toe passes the opposite leg’s midpoint between the ankle and knee.

##### Maximum Lateral Trunk Shift (#17)

The EVGS instructions for maximum lateral trunk shift are not machine-friendly because the scale’s description only refers to “marked,” “reduced,” or “moderate” trunk shift, with no threshold values provided to distinguish between the various conditions. The proposed approach for determining lateral trunk shift involves computing the angle between the trunk axis and the image coordinate’s y-axis in the coronal plane. Angle thresholds at which the trunk shift are normal, moderate, and marked and were determined using the development dataset. Videos from the coronal view that displayed any of the three conditions (normal, mild trunk shift, marked trunk shift) were analyzed. After analysis, maximum lateral trunk shift was considered normal if the angle was between 0° and 5°, reduced if the angle was less than zero, moderate if the trunk angle was between 6° and 15°, and severely inclined if the trunk angle was greater than 15°.

##### Knee Progression Angle (#8)

Knee progression angle is highly subjective and depends on visual cues. This parameter determines if the kneecap is visible. EVGS instructions are insufficient to determine the knee progression angle using only OpenPose keypoints. The proposed method of determining knee progression angle involves obtaining the ankle angle and establishing threshold values using the development dataset. Following video analysis, normal was chosen as an ankle angle between −25° and 25°, internal rotation as an ankle angle more than 25°, and external rotation as an ankle angle less than −25°.

##### Hindfoot Valgus/Varus (#4)

Hindfoot valgus/varus was calculated using a line connecting the ankle (KP11 and KP14) and heel keypoints (KP21 and KP24) and the image coordinate’s y-axis in the coronal plane.

The algorithmic implementation of the Edinburgh Visual Gait Score is presented in Pseudocode in Appendix B.

### 2.2. Algorithm Evaluation Methodology

Clinical implementation of EVGS is human-scored. To evaluate the proposed new EVGS algorithmic approach, the algorithmic scoring was evaluated by comparison to scoring by a panel of reviewers.

To evaluate the automated EVGS analysis system, a set of videos was collected that represented gait conditions for each EVGS parameter and result. This also allowed for a thorough evaluation of system performance, because the videos could be used to test various gait patterns.

Three healthy people with a good understanding of gait volunteered for this study. Participants were instructed to wear running shoes; tightly fitting, brightly colored clothes; and shorts with patellae (kneecaps) visible. Participants were female, 20 to 25 years, and with no issues affecting walking. All participants provided informed consent, and the study was approved by the University of Ottawa Research Ethics Board.

Before video recording, gait characteristics mentioned in the EVGS scale were explained to participants so that they could recreate these conditions. Nine gait sets were collected for the sagittal view: healthy, hip, knee, trunk, ankle, pelvis, foot position, heel lift, and foot clearance (Table 3). Five gait sets were collected for the coronal view: healthy, knee, trunk, foot, and pelvis (Table 4). Thirty-seven walking trials were collected, one for each condition and two for normal gait. This approach allowed for a comprehensive examination of different gait conditions and helped in the evaluation of algorithms for automatically calculating EVGS.

Data were collected using an iPhone 13 pro with a 6.1-inch display and 2532 × 1170 rear camera pixel resolution. The smartphone was handheld to replicate situations without a tripod or other support. Three individuals were involved in each recording process: volunteer being recorded, phone video recording operator, and assistant monitoring the recording. The operator was instructed to maintain a steady hand and hold the camera in portrait orientation at approximately neck level during the entire trial to ensure that the participant’s entire body was captured in the video. The camera was oriented parallel to the plane being captured. For a coronal view, the operator stood in front of the participant. A satisfactory video had the participant visible, balanced lighting, and no images, objects, or people in the background that may be mistaken for the participant. A brief description, diagram, or video of each gait pattern was provided before the video trial to ensure that the participants understood and interpreted the walking pattern correctly. Additionally, an EVGS manual was provided for the participants to read before the video session to help reduce bias that may be introduced by any demonstration of the gait pattern.

For each trial, participants walked 10 m at a comfortable self-selected speed in an obstacle-free and flat hallway. Following the initial 10 m walk, individuals took a brief two-second break, turned 180° in their chosen direction, took a brief two-second break, and then resumed straight walking. Each participant completed 37 walking trials, resulting in 111 videos being collected.

Each video was trimmed at the start and end to eliminate frames where the person was not on camera. Sagittal view videos were divided into left-to-right and right-to-left directions, while coronal view videos were divided into front view and rear view. Video quality was checked to ensure that it met the required standards. Videos that were not taken correctly were removed and recaptured. After preprocessing, 216 videos were available for analysis.

#### 2.2.1. Reviewers and Training

Five University of Ottawa students volunteered to score videos, each person with a sufficient understanding of gait events. Before evaluating the videos, all reviewers were briefed on normal gait kinematics, gait phases, and methodologies for recording and reviewing gait recordings. Additionally, the purpose and background of the EVGS were discussed, and gait analysis using the EVGS was demonstrated.

For training, the reviewers manually scored an example video using the EVGS and then compared their results to gain a better understanding. The reviewers then applied EVGS to evaluate the videos independently, without consulting one another. Slow motion and freeze-frame playback were used with video player software. Reviewers did not have time restrictions and could work at their own pace while reviewing.

#### 2.2.2. Algorithm Evaluation

To validate the approach to automatically calculating EVGS results, independent evaluations were completed for stride detection and EVGS results.

For foot event ground truth, one reviewer manually identified foot events in each video. The ground truth was then manually labeled for coronal/sagittal view, motion direction, foot strike and foot off, mid-midstance, and the total number of strides in each video. For EVGS algorithm evaluation, each video was scored by at least two reviewers. The reviewers used the EVGS to evaluate the videos independently, without consulting one another. Each reviewer was unaware of the other reviewer’s scoring.

Each reviewer completed an EVGS form containing all the EVGS parameters for each leg. The reviewers evaluated the entire gait cycle, including multiple strides, for each leg in each video. Only the most frequent score for each parameter for each leg in each video was considered as the final score. The use of the most frequent score also helped reduce potential biases introduced by evaluating only a single stride. The videos were rated between 0 and 2, with 0 denoting normal performance and 2 denoting highly abnormal, as specified in EVGS. Reviewers were instructed to assess joint angles using their eyes alone, without using any software or other tools, to replicate the instructions described in EVGS.

Once the ground truth for coronal/sagittal view, motion direction, foot strike and foot off, mid-midstance, the total number of strides, and the EVGS results were provided by the reviewers for each video, the algorithmic results and EVGS results were compared with the reviewers’ results. When using a human-scored scale, such as the EVGS, it is important to assess the degree of agreement or disagreement between different reviewers. Therefore, Pearson correlations were calculated for scores assigned by different reviewers, to determine the degree of consistency. Correlations were also used as a metric to compare algorithmic and reviewer results, with correlations interpreted as very high (0.9–1.0), high (0.7–0.9), moderate (0.5–0.7), low (0.3–0.5), and negligible (0.0–0.3).

## 3. Results

### 3.1. Coronal/Sagittal View Detection

To assess sagittal or coronal view identification, algorithmic findings and ground truth were compared. Table 5 displays the sagittal or coronal view (number of videos) classification confusion matrices. Accuracy was 96.3%, sensitivity was 93.1%, specificity was 98.4%, precision was 97.6%, and the F1-score was 0.95.

Algorithm accuracy was acceptable. The algorithm failed when multiple people were in the video or when a person unexpectedly turned towards the camera while walking in the sagittal view.

### 3.2. Direction of Motion Detection

Table 6 and Table 7 show confusion matrices for detecting the direction of motion (number of videos). For the coronal view, accuracy was 92.8%, sensitivity was 90.9%, specificity was 95.0%, precision was 95.2%, and the F1-score was 0.93. For the sagittal view, accuracy was 92.4%, sensitivity was 93.7%, specificity was 91.1%, precision was 90.9%, and the F1-score was 0.92.

The algorithm correctly determined direction in the majority of videos but failed when a person’s reflection was visible on the wall, when numerous people were in the video, when the camera moved as the person moved (both small and large camera movements), or when the participant was not in the frame the entire time.

### 3.3. Foot Strike, Foot off, and Mid-Midstance Detection

Algorithmically determined foot strike, foot off, and mid-midstance were compared to manually labelled ground truth. Each video was classified into three categories based on the discrepancy between the identified frame number and the actual frame number: two frames or less discrepancy, two to five frames discrepancy, and more than five frames discrepancy. Each stride in a video was categorized, and then the most frequently occurring category was assigned to the video.

**Figure 10:**
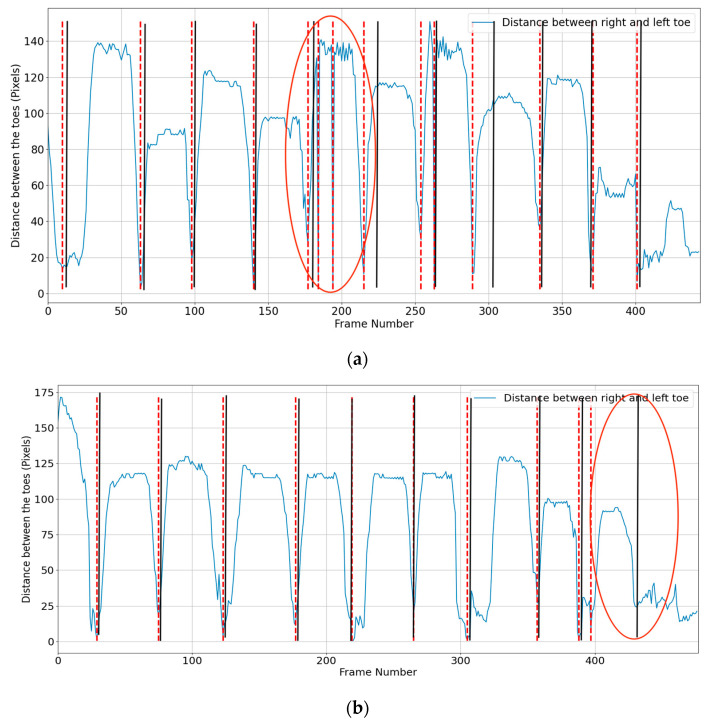
Example where the algorithm detects additional mid-midstance (**a**) but fails to detect a mid-midstance (**b**) (shown by ellipse region). The red dashed line is algorithm’s estimated mid-midstance. Solid black lines are ground truth for mid-midstance.

Additional foot events may occasionally be detected by the algorithm, while at other times, the algorithm may not detect an event (Figure 10). These circumstances occurred rarely (5 of 100 videos) when the OpenPose keypoints abruptly swapped between legs.

Table 8 shows accuracy results for foot strike, foot off, and mid-midstance.

### 3.4. Number of Strides

The difference between the number of strides detected by the algorithm and the number of strides detected by the reviewer are displayed in Table 9. Each video was classified into three categories based on the difference between the identified number of strides and the actual number of strides: two strides or less, two to five strides, and more than five strides.

### 3.5. EVGS Coronal Videos

**Figure 11:**
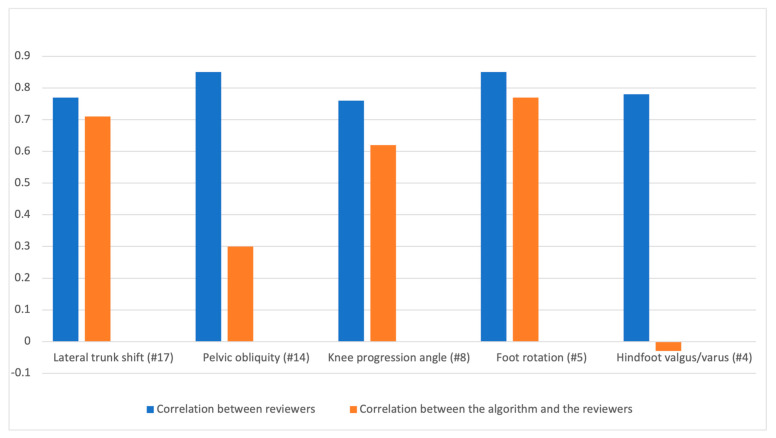
Reviewer correlation and correlation between the algorithm and the reviewers for coronal plane parameters.

The EVGS results were computed for five coronal view parameters. Pearson correlation analyses assessed the relationship between reviewer 1 and reviewer 2, reviewer 1 with the algorithm, and reviewer 2 with the algorithm, and for both legs and for each gait set. The EVGS result correlations between reviewers (R1, R2) and the algorithm for right and left legs for different gait sets are shown in Figure 11. The tables of results are in Appendix A.

### 3.6. EVGS Sagittal Videos

**Figure 12:**
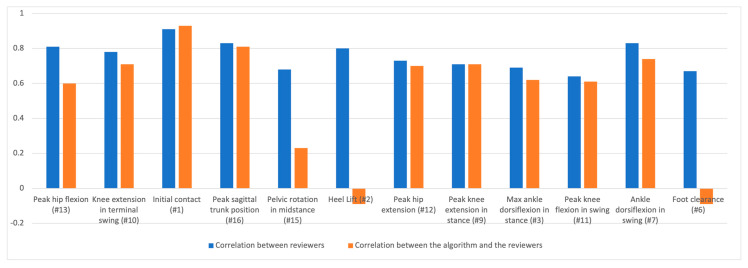
Reviewer correlation and correlation between the algorithm and reviewers for sagittal plane parameters.

The sagittal view had nine gait sets. The EVGS result correlations between reviewers (R1, R2) and the algorithm for right and left legs for different gait sets are shown in Figure 12. The tables of results are in Appendix A.

The results for each EVGS parameter are summarized in Table 10, using the overall average correlation between reviewers and the algorithm.

## 4. Discussion

The discussion is divided into subsections for coronal view parameters and sagittal view parameters.

### 4.1. Coronal View Parameters

Based on coronal view analysis across different gait sets, all five coronal parameters had high correlations among reviewers (r = 0.7 to 0.9). The literature on the EVGS interrater reliability found that the level of agreement between reviewers varied for each parameter [34]. The knee progression angle showed a high level of agreement, with an 81% agreement rate among reviewers, while the other parameters had a moderate level of agreement (r = 0.5–0.7). Although there were differences between the correlation coefficients in this research and the level of agreement in the literature, the overall range of agreement among reviewers for each gait parameter was consistent between this study and the literature.

**Figure 13:**
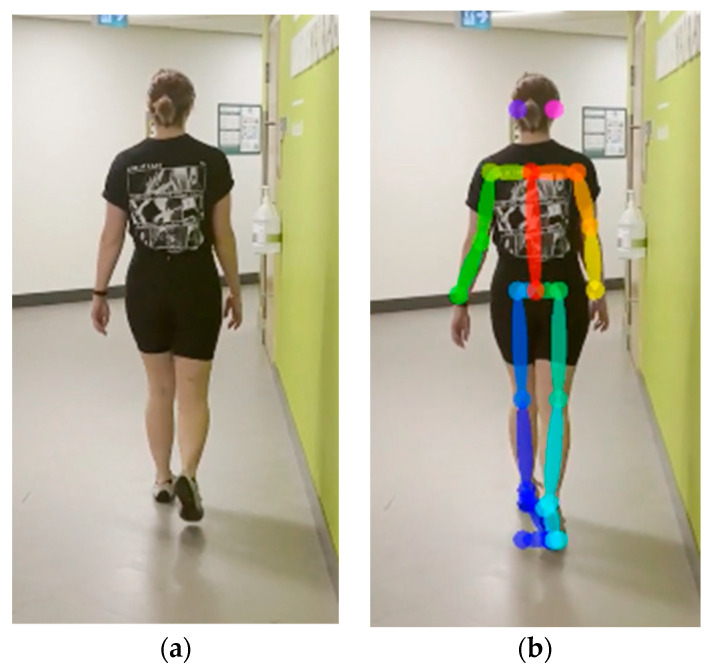
An example where OpenPose fails to recognize heel, ankle, big toe, and small toe accurately. Image without keypoints (**a**) and image with keypoints errors at the ankle and foot (**b**).

Foot rotation and lateral trunk shift showed high correlations between the algorithm and reviewers, while the knee progression angle had moderate correlations. Pelvic obliquity had low correlations, and the hindfoot valgus/varus parameter showed negligible correlations between the algorithm and reviewers. Keypoints on the ankle and heel that are near each other in a narrow area and far from the camera are more likely to be occluded during walking, which helps to explain this result. Figure 13 depicts foot keypoints as the person walks away from the camera. Since the two legs are close together, the foot keypoints for the left leg are not accurately detected. Possible improvements to address these issues could include using the knowledge that the stance foot should not move (i.e., marker location fixed) when not “toe walking”, thereby helping to avoid confusion when the swing foot passes close to the stance foot. Additional model training for these situations could also help improve OpenPose keypoint determination.

Pelvic obliquity involves movement of the entire pelvis segment. Hip keypoints were used as a surrogate measure of pelvic movement, because they are a part of the pelvic region but may not represent the entire pelvic segment; therefore, lower correlations between the algorithm and reviewer were anticipated. The difference was also attributed to the reviewer looking at the entire pelvis when making their assessment, which differs from the algorithm only using hip keypoints. In future, improved AI models that estimate depth (i.e., 3D coordinates for each keypoint) could provide additional information to improve outcomes when using the hips as a surrogate marker.

With the exception of hindfoot valgus and varus, all parameters demonstrated strong correlations between reviewers and the algorithm for the healthy gait set. The knee progression angle had a stronger correlation within the knee gait set, while the foot progression angle and lateral trunk shift had the strongest correlation within the foot gait set.

All correlations between the algorithm and the reviewer were lower than between the review outcomes. As mentioned previously, this difference can be attributed to surrogate measures from the OpenPose BODY25 keypoints. In addition, if the camera is not parallel to the coronal plane and oriented vertically, parallax effects may not accurately depict the body keypoints, and phone orientation can affect measures where the phone axis is assumed to be aligned to the ground. Keypoint detection errors can also occur for body parts that are closer to the camera. Future research could include phone sensor data to enable video frame transformation to gravity, thereby helping to provide a consistent ground plane.

### 4.2. Sagittal View Parameters

Based on sagittal view analysis, the agreement between reviewers was very high for initial contact, followed by six parameters with high correlations, namely peak sagittal trunk position (#16), ankle dorsiflexion in swing (#7), peak hip flexion (#13), heel lift (#2), knee extension in terminal swing (#10), and peak hip extension (#12). Four parameters had moderate correlations (max. ankle dorsiflexion in stance (#3), pelvic rotation in midstance (#15), foot clearance (#6), and peak knee flexion in swing (#11)). The literature on the interrater reliability of sagittal view gait parameters reported that initial contact had an agreement rate of 90%, while foot clearance and heel lift had agreement rates of 82–83% [34]. Knee extension in terminal swing had an agreement rate of 62%, and knee peak flexion in swing had an agreement rate of 69%. For both the research results and the literature, initial contact was the most consistent parameter between raters for sagittal view gait parameters. Other parameters, such as trunk position, ankle dorsiflexion in swing, foot clearance, and heel lift, also had high levels of agreement between reviewers. However, knee flexion, knee extension in terminal swing, and knee peak flexion in swing had lower levels of agreement.

The foot position parameter had a very high correlation between the algorithm and the reviewer. Five parameters had a high correlation: peak sagittal trunk position (#16), ankle dorsiflexion in swing (#7), knee extension in terminal swing (#10), peak knee extension in stance (#9), and peak hip extension (#12). Three parameters had a moderate correlation: max. ankle dorsiflexion in stance (#3), peak knee flexion in swing (#11), and peak hip flexion (#13). Finally, three parameters had a negligible correlation: pelvic rotation in midstance (#15), heel lift (#2), and foot clearance (#6). Foot parameter correlations were close to zero, indicating no relationship between the algorithm and reviewer scores. Foot parameters are the keypoints farthest from the cameras, can be occluded during walking, can rely on the floor plane being correct, and as a terminal segment, can have poorer OpenPose performance. Pelvic parameters had lower correlations (r = 0.23), since the hip keypoints were used as a surrogate measure of pelvic movement. The difference could also relate to the reviewer’s assessment of the complete pelvis as opposed to the algorithm’s use of only the hip keypoints. In future research, estimating the keypoint depth coordinate could help improve the pelvic rotation scoring.

Knee flexion and ankle dorsiflexion in swing had stronger correlations between the algorithm and reviewers when using the foot position gait set. Trunk position had a better correlation for the knee gait set. In contrast, peak hip flexion and ankle dorsiflexion in swing had the strongest correlations when using the ankle gait set. The results of our research showed that ankle dorsiflexion had a stronger correlation with the foot and ankle gait dataset, which was specifically designed to focus on foot movements during gait. This may be due to the fact that the foot movements are more prominent in this gait dataset, making it easier for both reviewers and the algorithm to accurately detect and agree on the presence and extent of ankle dorsiflexion.

For peak hip flexion correlation, the between reviewers’ results were higher than the reviewers and algorithm correlations. This difference can be attributed to small differences in angle measurements leading to different scores. For example, a score of 0 corresponds to a peak hip flexion angle between 25° and 45°, while a score of 1 corresponds to angles between 45° and 60°. Reviewers may not accurately detect small angle changes, such as differences between 44° and 46°, leading to discrepancies in their assessments. In contrast, the algorithm assigns scores using the measured angle. Therefore, the algorithm provides a more objective and consistent assessment of the peak hip flexion parameter compared to the subjective assessments of human reviewers.

Foot strike and foot-off detection are crucial for the heel lift score. Even a small frame change affects this parameter. Consider, for instance, a scenario in which the opposite leg foot strike occurs at frame 53 and foot off occurs at frame 52. In this case, the patient would have a normal heel lift score (score 0). The stride detection algorithm would identify the foot strike at frame 53 and the foot off at 54. Foot event frames could be within the permitted range of 2, which is used for stride detection. The condition would, therefore, be considered delayed, and a score of one would be assigned. This demonstrates how even a small variation of 1 frame would substantially affect the heel lift parameter.

**Figure 14:**
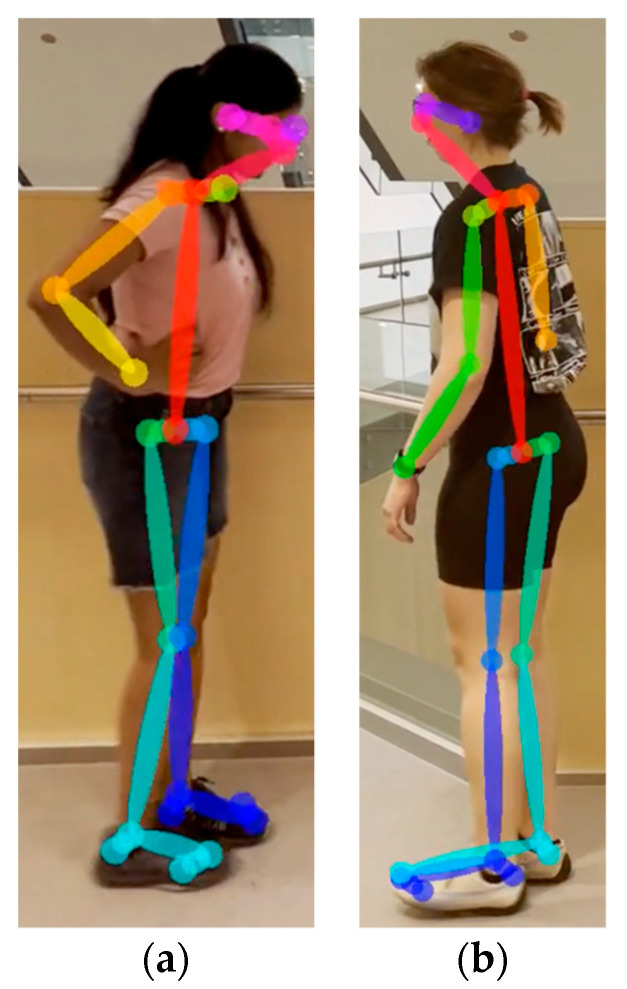
(**a**) No clearance. (**b**) Reduced clearance.

For foot clearance, reviewers scored Figure 14a as 2, “No clearance”. The person in Figure 14b was scored as 1, “Reduced clearance”. However, the algorithm categorized Figure 14a as “Reduced clearance” and Figure 14b as “No clearance”, as the keypoints were clustered in a small area, and there was no data for the floor axis. Considering that OpenPose does not locate foot keypoints at the shoe insole level and handheld smartphone video can lead to variable floor plane estimation, foot clearance and heel lift parameters will be difficult to achieve without further research. Potential improvements could involve using phone orientation data to correct the floor plane and continuing to develop the foot keypoint identification aspect of pose estimation models.

Algorithm performance varied across the different parameters. For the foot progression angle (#5), both the correlation between reviewers and the correlation between reviewers and the algorithm were high, indicating that the algorithm performed well in identifying this parameter. The algorithm performed reasonably well for the knee progression angle (#8) and lateral trunk shift (#17), with moderate and high agreement between reviewers and the algorithm, respectively. The algorithm’s performance was poor and negligible for pelvic obliquity (#14) and hindfoot valgus/varus (#4) in the coronal view compared to human reviewers. The correlation between the algorithm and each reviewer was moderate for pelvic obliquity and negligible for hindfoot valgus/varus, indicating the algorithm struggled to accurately identify these parameters in a way that aligned with human judgment. The correlation between the two reviewers for hindfoot valgus/varus was also negligible compared to other coronal parameters, which may have contributed to the algorithm’s lower performance.

## 5. Conclusions

This research proposes an automated approach for using the Edinburgh Visual Gait Score (EVGS) with handheld smartphone video. The results show that the algorithmic stride detection component works better for sagittal videos than coronal videos, and the EVGS performance varied across different parameters. Of the seventeen EVGS parameters, correlations between human reviewers and the algorithm were very high for one parameter, high for seven parameters, moderate for four parameters, low for one parameter, and negligible for four parameters. Based on these results, automated EVGS processing could now be used for 12 parameters, but more research is needed to properly classify pelvic rotation in midstance (#15), heel lift (#2), foot clearance in swing (#6), maximum pelvic obliquity in midstance (#14), and hindfoot valgus/varus (#4).

This research demonstrates the feasibility of using videos recorded using a handheld smartphone camera, processed by pose estimation models, and scored by rule-based algorithms. The proposed approach has the potential to enhance gait analysis accessibility and facilitate continuous monitoring of patients outside the clinic.

Future research should involve capturing smartphone orientation, accelerometer, and gyroscope data while recording videos and incorporating this information into the stride identification and EVGS algorithms for results, especially for foot-related parameters. To improve the accuracy of patient gait pattern capture, future research could gather data from more strides to recognize more foot events and minimize the effect of outliers. Additionally, machine learning algorithms could be implemented to potentially improve the automatic scoring of foot event and stride frame identification. An app for smartphone-based gait analysis could be developed, enabling the automatic acquisition of the Edinburgh Visual Gait Score (EVGS) after capturing patient video with the app and thereby enabling evidence-based decision-making during a patient encounter.

## Figures and Tables

**Table 1:** Pose estimation models.

	DeepPose	HyperPose	BlazePose	OpenPose
Skeleton model	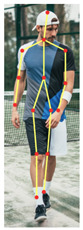	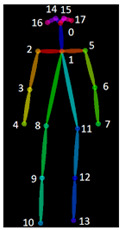	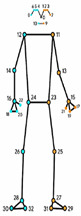	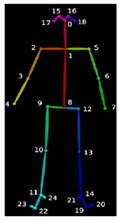
Reference	[30]	[24,28]	[25,29]	[26,28,29,31]
Neck/ trunk start	Yes	Yes	No	Yes
Midhip/trunk ending	No	No	No	Yes
Hip	Yes	Yes	Yes	Yes
Knee	Yes	Yes	Yes	Yes
Ankle	Yes	Yes	Yes	Yes
Heel	No	No	Yes	Yes
Toes	No	No	Yes	Yes

**Table 2:** List of EVGS parameters.

	Foot Events and Gait Phases	EVGS Parameters
Sagittal view	Initial contact/Terminal swing	Peak hip flexion in swing (#13)
		Knee extension in terminal swing (#10)
		Initial contact (#1)
	Midstance	Peak sagittal trunk position (#16)
		Pelvic rotation in midstance (#15)
		Heel lift (#2)
	Terminal stance	Peak hip extension in stance (#12)
		Peak knee extension in stance (#9)
		Max. ankle dorsiflexion in stance (#3)
	Midswing	Peak knee flexion in swing (#11)
		Maximum ankle dorsiflexion in swing (#7)
		Foot clearance in swing (#6)
Coronal view	Midstance	Maximum lateral shift of trunk (#17)
		Maximum pelvic obliquity in stance (#14)
		Knee progression angle (#8)
		Foot rotation (#5)
		Hindfoot valgus/varus (#4)

**Table 3:** Gait sets in sagittal view.

Gait Set	Characteristics	Description
Healthy	Normal	Walk normally
Hip	Flexion	At initial contact, walk with hips flexed
	Extension	At terminal stance, walk with hips extended
Knee	Flexion	At initial contact and midswing, walk with knee flexed
	Extension	At initial contact and terminal stance, walk with knee extended
Ankle	Dorsiflexion	During terminal stance and midswing, walk with ankle dorsiflexed
	Plantarflexion	During terminal stance and midswing, walk with ankle plantarflexed
Trunk	Reduced	Walk with trunk more than 5° backward
	Moderate	Lean forward 6° to 15° while walking
	Marked	Lean more than 15° forward while walking
Pelvis	Protraction	Walk with pelvic region extended more than 10°
	Retraction	Retract pelvic region more than 5° when walking
Foot position	Toe contact	Make initial contact with the floor with toes
	Heel contact	Make initial contact with the floor with heel
	Flat foot contact	Make initial contact with the ground with foot flat
Heel lift	No forefoot contact	Walk on heel
	No heel contact	Walk on toes
	Early	Raise your heel off the ground before opposing foot aligns with stance foot
	Delayed	Raise your heel from the ground after the opposing foot has contacted the ground
Foot clearance	High steps	Lift knees high in the air as you walk
	Reduced clearance	Do not completely lift foot off the ground when walking. Either the heel or toe is still in contact with the ground
	No clearance	Do not take feet off the ground. Drag feet

**Table 4:** Gait sets in coronal view.

Gait Set	Condition	Description
Healthy	Normal	Walk normally
Knee	External: Full kneecap	Walk with knees externally rotated and whole kneecaps visible
	External: Part kneecap	Walk with knees externally rotated and part of the kneecaps visible
	Internal: Full kneecap	Walk with knees internally rotated and whole kneecaps visible
	Internal: Part kneecap	Walk with knees internally rotated and part of the kneecaps visible
Foot	External: Moderate	Walk with feet 21° to 40° externally rotated
	External: Marked	Walk with feet turned externally by more than 40°
	Internal: Marked	Walk with feet 1° to 25° internally rotated
	Internal: Moderate	Walk with feet internally rotated by greater than 25°
Pelvis	Up	Lift one hip over the other by more than 5°
	Down	Lower one hip more than 1° while raising the other
Trunk	Reduced	During midstance, walk with trunk tilted toward the opposing leg
	Moderate	Tilt trunk more than 25 mm toward the stance leg at midstance
	Marked	At midstance, lean trunk substantially towards the stance leg

**Table 5:** Confusion matrix for coronal/sagittal view identification.

	Algorithm	
Ground Truth	Coronal	Sagittal
Coronal	82	2
Sagittal	6	126

**Table 6:** Confusion matrix for detecting direction of motion (coronal view).

	Algorithm	
Ground Truth	Coronal	Sagittal
Coronal	40	2
Sagittal	4	38

**Table 7:** Confusion matrix for detecting direction of motion (sagittal view).

	Algorithm	
Ground Truth	Coronal	Sagittal
Coronal	60	6
Sagittal	4	62

**Table 8:** Foot strike accuracy categories for sagittal and coronal views (number of videos).

		Differ by More than Five Frames	Differ by Two to Five Frames	Differ by Two Frames or Less
Foot strike	Coronal	22	27	35
	Sagittal	20	91	21
Foot off	Coronal	20	33	31
	Sagittal	19	87	26
Mid-midstance	Coronal	18	24	42
	Sagittal	4	1	127

**Table 9:** Difference between the number of strides detected by the algorithm and the number of strides detected by the reviewer (number of videos).

	Differ by More than Five Strides	Differ by Two to Five Strides	Differ by Two Strides or Less
Coronal	3	4	77
Sagittal	3	3	126

**Table 10:** Correlation interpretation between reviewers (R1 and R2) and algorithm.

	Parameter	Quality
Coronal parameters	Foot rotation (#5)	High
	Maximum lateral trunk shift (#17)	High
	Knee progression angle (#8)	Moderate
	Maximum pelvic obliquity in midstance (#14)	Low
	Hindfoot valgus/varus (#4)	Negligible
Sagittal parameters	Initial contact (#1)	Very high
	Peak sagittal trunk position (#16)	High
	Maximum ankle dorsiflexion in swing (#7)	High
	Knee extension in terminal swing (#10)	High
	Peak knee extension in stance (#9)	High
	Peak hip extension in stance (#12)	High
	Maximum ankle dorsiflexion in stance (#3)	Moderate
	Peak knee flexion in swing (#11)	Moderate
	Peak hip flexion in swing (#13)	Moderate
	Pelvic rotation in midstance (#15)	Negligible
	Heel lift (#2)	Negligible
	Foot clearance in swing (#6)	Negligible

## Data Availability

Not applicable.

## Appendix A

**Table A1:** EVGS correlations between reviewers (R1, R2) and the algorithm for healthy gait set coronal parameters.

Parameter	R1 and R2		R1 and Algorithm		R2 and Algorithm	
	Right	Left	Right	Left	Right	Left
Lateral trunk shift (#17)	1.00	1.00	0.84	0.61	0.84	0.61
Pelvic obliquity (#14)	0.92	1.00	0.92	1.00	0.84	1.00
Knee progression angle (#8)	1.00	1.00	0.92	1.00	0.92	1.00
Foot progression angle (#5)	1.00	1.00	1.00	0.83	1.00	0.83
Hindfoot valgus/varus (#4)	1.00	1.00	−0.61	−0.61	−0.61	−0.61

**Table A2:** EVGS correlations between reviewers (R1, R2) and algorithm for knee gait set coronal parameters.

Parameter	R1 and R2		R1 and Algorithm		R2 and Algorithm	
	Right	Left	Right	Left	Right	Left
Lateral trunk shift (#17)	0.52	0.77	0.77	0.62	0.67	0.81
Pelvic obliquity (#14)	0.52	1.00	−0.26	−0.26	−0.13	−0.26
Knee progression angle (#8)	0.45	0.67	0.70	0.92	0.87	0.87
Foot progression angle (#5)	0.92	1.00	0.77	0.83	0.83	0.64
Hindfoot valgus/varus (#4)	0.89	0.67	0.20	0.20	−0.04	0.30

**Table A3:** EVGS correlations between reviewers (R1, R2) and the algorithm for foot gait set coronal parameters.

Parameter	R1 and R2		R1 and Algorithm		R2 and Algorithm	
	Right	Left	Right	Left	Right	Left
Lateral trunk shift (#17)	0.90	1.00	1.00	1.00	0.87	1.00
Pelvic obliquity (#14)	0.82	1.00	−0.58	0.58	0.71	0.58
Knee progression angle (#8)	0.58	0.58	0.58	0.58	0.33	1.00
Foot progression angle (#5)	1.00	1.00	1.00	0.90	1.00	0.90
Hindfoot valgus/varus (#4)	0.58	0.58	0.33	0.33	−0.58	−0.58

**Table A4:** EVGS correlations between reviewers (R1, R2) and algorithm for pelvis gait set coronal parameters.

Parameter	R1 and R2		R1 and Algorithm		R2 and Algorithm	
	Right	Left	Right	Left	Right	Left
Lateral trunk shift (#17)	0.70	0.29	0.77	0.45	0.54	0.22
Pelvic obliquity (#14)	0.92	0.67	0.60	−0.41	0.45	−0.31
Knee progression angle (#8)	0.88	0.71	0.45	0.10	0.22	0.49
Foot progression angle (#5)	0.49	0.61	0.27	0.71	0.91	0.06
Hindfoot valgus/varus (#4)	0.92	0.84	−0.38	0.38	0.54	0.38

**Table A5:** EVGS correlations between reviewers (R1, R2) and algorithm for trunk gait set coronal parameters.

Parameter	R1 and R2		R1 and Algorithm		R2 and Algorithm	
	Right	Left	Right	Left	Right	Left
Lateral trunk shift (#17)	0.72	0.84	0.75	0.64	0.60	0.50
Pelvic obliquity (#14)	0.87	0.78	0.10	0.64	−0.12	0.82
Knee progression angle (#8)	0.91	0.78	0.33	0.41	0.28	0.41
Foot progression angle (#5)	0.60	0.92	0.77	0.83	0.43	0.91
Hindfoot valgus/varus (#4)	0.68	0.68	0.22	−0.03	0.32	0.22

**Table A6:** The overall average correlation between reviewers and algorithm of entire dataset, as well as the average correlation of reviewers for each coronal parameter.

Parameters	R1 and R2	R1, R2 and Algorithm		
		Right	Left	Average
Lateral trunk shift (#17)	0.77	0.77	0.65	0.71
Pelvic obliquity (#14)	0.85	0.25	0.34	0.30
Knee progression angle (#8)	0.76	0.56	0.68	0.62
Foot rotation (#5)	0.85	0.80	0.74	0.77
Hindfoot valgus/varus (#4)	0.78	−0.06	0.00	−0.03

**Table A7:** EVGS correlations between reviewers (R1, R2) and algorithm for healthy gait sagittal parameters.

Parameters	R1 and R2		R1 and Algorithm		R2 and Algorithm	
	Right	Left	Right	Left	Right	Left
Peak hip flexion (#13)	1.00	1.00	1.00	1.00	1.00	1.00
Knee extension in terminal swing (#10)	1.00	1.00	1.00	1.00	1.00	1.00
Initial contact (#1)	1.00	1.00	1.00	1.00	1.00	1.00
Peak sagittal trunk position (#16)	1.00	1.00	1.00	1.00	1.00	1.00
Pelvic rotation in midstance (#15)	1.00	1.00	0.69	0.71	0.92	0.98
Heel lift (#2)	1.00	1.00	0.41	0.41	0.41	0.41
Peak hip extension (#12)	1.00	1.00	1.00	0.91	1.00	0.91
Peak knee extension in stance (#9)	1.00	1.00	0.92	0.87	0.92	0.87
Max. ankle dorsiflexion in stance (#3)	1.00	1.00	1.00	0.75	1.00	0.75
Peak knee flexion in swing (#11)	1.00	1.00	1.00	1.00	1.00	1.00
Ankle dorsiflexion in swing (#7)	1.00	1.00	0.92	1.00	0.92	1.00
Foot clearance (#6)	1.00	1.00	−0.17	−0.24	0.17	−0.24

**Table A8:** EVGS correlations between reviewers (R1, R2) and algorithm for hip set sagittal parameters.

Parameters	R1 and R2		R1 and Algorithm		R2 and Algorithm	
	Right	Left	Right	Left	Right	Left
Peak hip flexion (#13)	1.00	1.00	0.85	0.88	0.85	0.88
Knee extension in terminal swing (#10)	0.47	0.35	0.47	0.73	1.00	0.26
Initial contact (#1)	1.00	1.00	1.00	1.00	1.00	1.00
Peak sagittal trunk position (#16)	0.75	0.75	0.73	1.00	0.55	0.75
Pelvic rotation in midstance (#15)	1.00	0.91	0.73	0.93	0.73	0.88
Heel lift (#2)	0.75	0.75	0.47	0.35	0.35	0.47
Peak hip extension (#12)	1.00	0.89	1.00	0.91	1.00	0.77
Peak knee extension in stance (#9)	1.00	1.00	0.92	0.87	0.92	0.87
Max. ankle dorsiflexion in stance (#3)	0.68	0.68	0.55	0.75	0.37	0.11
Peak knee flexion in swing (#11)	0.55	0.55	0.55	0.73	1.00	0.40
Ankle dorsiflexion in swing (#7)	0.30	1.00	0.65	1.00	−0.26	1.00
Foot clearance (#6)	0.32	0.32	−0.11	0.24	−0.47	0.17

**Table A9:** EVGS correlations between reviewers (R1, R2) and algorithm for knee gait set sagittal parameters.

Parameters	R1 and R2		R1 and Algorithm		R2 and Algorithm	
	Right	Left	Right	Left	Right	Left
Peak hip flexion (#13)	1.00	1.00	0.65	0.00	0.65	0.00
Knee extension in terminal swing (#10)	0.63	0.86	0.63	0.61	1.00	0.84
Initial contact (#1)	1.00	1.00	1.00	1.00	1.00	1.00
Peak sagittal trunk position (#16)	1.00	1.00	0.71	1.00	0.71	1.00
Pelvic rotation in midstance (#15)	0.71	0.71	−0.24	0.22	−0.34	0.46
Heel lift (#2)	1.00	1.00	−0.32	−0.32	−0.32	−0.32
Peak hip extension (#12)	1.00	1.00	−0.20	1.00	−0.20	1.00
Peak knee extension in stance (#9)	1.00	1.00	1.00	0.88	1.00	0.88
Max. ankle dorsiflexion in stance (#3)	0.82	0.82	0.27	1.00	0.50	0.82
Peak knee flexion in swing (#11)	0.63	0.63	0.45	0.61	0.71	0.77
Ankle dorsiflexion in swing (#7)	0.89	0.89	1.00	0.20	0.89	0.45
Foot clearance (#6)	−0.29	1.00	0.00	0.20	0.56	0.20

**Table A10:** EVGS correlations between reviewers (R1, R2) and algorithm for ankle gait set sagittal parameters.

Parameters	R1 and R2		R1 and Algorithm		R2 and Algorithm	
	Right	Left	Right	Left	Right	Left
Peak hip flexion (#13)	1.00	1.00	1.00	1.00	1.00	1.00
Knee extension in terminal swing (#10)	1.00	1.00	1.00	0.77	1.00	0.77
Initial contact (#1)	1.00	1.00	1.00	1.00	1.00	1.00
Peak sagittal trunk position (#16)	1.00	0.71	0.71	1.00	0.71	0.71
Pelvic rotation in midstance (#15)	0.63	0.63	−0.43	0.71	−0.55	0.45
Heel lift (#2)	0.88	0.88	−0.80	−0.80	−0.88	−0.88
Peak hip extension (#12)	1.00	0.71	0.63	0.50	0.63	0.71
Peak knee extension in stance (#9)	0.84	0.58	0.77	0.61	0.43	0.71
Max. ankle dorsiflexion in stance (#3)	0.88	0.88	0.93	0.88	0.79	0.80
Peak knee flexion in swing (#11)	0.17	0.77	0.86	0.77	0.41	0.50
Ankle dorsiflexion in swing (#7)	1.00	1.00	1.00	1.00	1.00	1.00
Foot clearance (#6)	0.63	0.63	0.29	−0.25	0.00	−0.63

**Table A11:** EVGS correlations between reviewers (R1, R2) and algorithm for trunk gait set sagittal parameters.

Parameters	R1 and R2		R1 and Algorithm		R2 and Algorithm	
	Right	Left	Right	Left	Right	Left
Peak hip flexion (#13)	0.82	0.67	−0.45	0.15	−0.46	0.23
Knee extension in terminal swing (#10)	0.89	0.70	1.00	0.75	0.89	0.67
Initial contact (#1)	0.81	0.81	1.00	0.86	0.81	0.78
Peak sagittal trunk position (#16)	1.00	1.00	0.82	0.82	0.82	0.82
Pelvic rotation in midstance (#15)	0.88	0.88	−0.45	0.01	−0.32	−0.02
Heel lift (#2)	0.80	0.88	0.80	0.88	1.00	1.00
Peak hip extension (#12)	0.67	0.38	0.67	0.67	1.00	0.67
Peak knee extension in stance (#9)	0.68	0.50	0.75	0.90	0.75	0.43
Max. ankle dorsiflexion in stance (#3)	0.80	0.80	0.91	1.00	0.49	0.80
Peak knee flexion in swing (#11)	0.49	0.38	0.90	0.82	0.30	0.31
Ankle dorsiflexion in swing (#7)	1.00	1.00	1.00	0.67	1.00	0.67
Foot clearance (#6)	0.55	0.94	−0.22	−0.16	−0.23	−0.17

**Table A12:** EVGS correlations between reviewers (R1, R2) and algorithm for pelvis gait set sagittal parameters.

Parameters	R1 and R2		R1 and Algorithm		R2 and Algorithm	
	Right	Left	Right	Left	Right	Left
Peak hip flexion (#13)	0.61	0.17	0.58	−0.33	1.00	0.87
Knee extension in terminal swing (#10)	0.77	0.74	0.36	0.83	0.32	0.78
Initial contact (#1)	0.81	0.88	0.97	0.89	0.85	0.77
Peak sagittal trunk position (#16)	0.76	0.65	0.72	0.86	0.81	0.66
Pelvic rotation in midstance (#15)	0.56	0.43	0.22	0.27	−0.02	0.12
Heel lift (#2)	0.69	0.68	−0.59	−0.32	−0.22	−0.22
Peak hip extension (#12)	0.54	0.57	0.71	1.00	0.45	0.53
Peak knee extension in stance (#9)	0.52	0.54	0.77	0.35	0.64	0.55
Max. ankle dorsiflexion in stance (#3)	0.47	0.54	0.64	0.40	0.53	0.38
Peak knee flexion in swing (#11)	0.68	0.67	0.70	0.61	0.40	0.27
Ankle dorsiflexion in swing (#7)	0.69	0.75	0.79	0.51	0.58	0.73
Foot clearance (#6)	0.73	0.74	−0.33	0.10	−0.38	0.05

**Table A13:** EVGS correlations between reviewers (R1, R2) and algorithm for foot position gait set sagittal parameters.

Parameters	R1 and R2		R1 and Algorithm		R2 and Algorithm	
	Right	Left	Right	Left	Right	Left
Peak hip flexion (#13)	1.00	1.00	1.00	0.50	1.00	0.50
Knee extension in terminal swing (#10)	0.75	0.75	0.75	1.00	0.72	0.75
Initial contact (#1)	1.00	1.00	1.00	0.92	1.00	0.92
Peak sagittal trunk position (#16)	0.94	0.94	0.80	0.74	0.75	0.81
Pelvic rotation in midstance (#15)	0.77	0.70	0.67	0.52	0.29	0.60
Heel lift (#2)	0.93	0.93	−0.92	−0.58	−0.94	−0.60
Peak hip extension (#12)	0.80	0.80	1.00	1.00	0.80	0.80
Peak knee extension in stance (#9)	1.00	1.00	0.76	0.89	0.76	0.89
Max. ankle dorsiflexion in stance (#3)	0.45	0.45	0.90	0.00	0.43	0.44
Peak knee flexion in swing (#11)	1.00	1.00	0.87	0.94	0.87	0.94
Ankle dorsiflexion in swing (#7)	1.00	1.00	1.00	0.90	1.00	0.90
Foot clearance (#6)	0.76	0.76	−0.22	0.50	−0.22	0.66

**Table A14:** EVGS correlations between reviewers (R1, R2) and algorithm for heel lift gait set sagittal parameters.

Parameters	R1 and R2		R1 and Algorithm		R2 and Algorithm	
	Right	Left	Right	Left	Right	Left
Peak hip flexion (#13)	0.52	0.76	0.18	0.53	−0.08	0.41
Knee extension in terminal swing (#10)	0.69	0.69	0.23	0.84	0.36	0.76
Initial contact (#1)	0.82	1.00	1.00	0.82	0.82	0.82
Peak sagittal trunk position (#16)	0.67	0.30	0.67	1.00	1.00	0.30
Pelvic rotation in midstance (#15)	0.82	0.82	−0.26	0.22	−0.16	0.27
Heel lift (#2)	0.90	0.90	−0.69	0.00	0.72	0.05
Peak hip extension (#12)	0.53	0.53	0.36	1.00	0.43	0.53
Peak knee extension in stance (#9)	0.76	0.76	0.90	0.35	0.88	0.23
Max. ankle dorsiflexion in stance (#3)	0.55	0.67	0.07	0.68	0.59	0.43
Peak knee flexion in swing (#11)	0.38	0.38	0.36	0.60	−0.26	−0.04
Ankle dorsiflexion in swing (#7)	0.83	0.76	0.54	0.76	0.32	1.00
Foot clearance (#6)	0.67	0.67	−0.27	0.11	−0.41	0.17

**Table A15:** EVGS correlations between reviewers (R1, R2) and algorithm for foot clearance gait set sagittal parameters.

Parameters	R1 and R2		R1 and Algorithm		R2 and Algorithm	
	Right	Left	Right	Left	Right	Left
Peak hip flexion (#13)	0.76	0.31	0.89	0.59	0.88	0.85
Knee extension in terminal swing (#10)	0.87	0.78	0.10	0.64	−0.12	0.82
Initial contact (#1)	0.63	0.64	0.90	0.92	0.73	0.58
Peak sagittal trunk position (#16)	0.69	0.71	0.69	0.84	0.69	0.85
Pelvic rotation in midstance (#15)	0.08	−0.22	0.24	0.08	−0.20	−0.51
Heel lift (#2)	0.23	0.21	−0.17	−0.39	−0.43	−0.10
Peak hip extension (#12)	0.29	0.37	0.77	1.00	0.13	0.24
Peak knee extension in stance (#9)	−0.19	−0.15	0.64	−0.19	0.27	0.53
Max. ankle dorsiflexion in stance (#3)	0.41	0.51	0.96	0.52	0.55	0.26
Peak knee flexion in swing (#11)	0.65	0.63	0.88	0.27	0.60	−0.09
Ankle dorsiflexion in swing (#7)	0.25	0.49	0.82	−0.15	0.43	0.30
Foot clearance (#6)	0.77	0.81	−0.51	−0.30	−0.52	−0.68

**Table A16:** The overall average correlation between reviewers and algorithm, as well as the average correlation of reviewers for each sagittal parameter.

Parameters	R1 and R2	R1, R2, and Algorithm		
		Right	Left	Average
Peak hip flexion (#13)	0.81	0.64	0.56	0.60
Knee extension in terminal swing (#10)	0.78	0.65	0.77	0.71
Initial contact (#1)	0.91	0.95	0.90	0.93
Peak sagittal trunk position (#16)	0.83	0.77	0.84	0.81
Pelvic rotation in midstance (#15)	0.68	0.09	0.38	0.23
Heel lift (#2)	0.80	−0.12	−0.05	−0.09
Peak hip extension (#12)	0.73	0.62	0.79	0.70
Peak knee extension in stance (#9)	0.71	0.78	0.64	0.71
Max. ankle dorsiflexion in stance (#3)	0.69	0.64	0.60	0.62
Peak knee flexion in swing (#11)	0.64	0.64	0.58	0.61
Ankle dorsiflexion in swing (#7)	0.83	0.76	0.72	0.74
Foot clearance (#6)	0.67	−0.17	−0.01	−0.09

## Appendix B

Pseudocode for algorithmic implementation of EVGS

1. Obtain video from user and load it into the program
2. Apply OpenPose-BODY25 model to extract joint coordinates as keypoints for each video frame and store in variable KP_raw1,KP_raw2,…..KP_raw24
3. Filter the keypoint data using a zero-phase, dual-pass, second-order 12 Hz Butterworth filter (filtered_keypoints: KP_filter1,KP_filter2,…..KP_filter24)
4. Remove keypoints with scores below a 10% threshold (threshold = 0.1)

  For each keypoint in filtered_keypoints:

    If keypoint score < threshold, remove keypoint.

5. Fill gaps of five frames or fewer using cubic spline interpolation (interpolated_keypoints: KP1,KP2,…..KP24)
6. **Determine the orientation of the video:**

  trunk_length_diff = abs(euclidean_distance(KP0, KP8)[0] − euclidean_distance(KP0, KP8)[Last frame (L) − 1])

If trunk_length_diff < threshold (99 frames): video_orientation = “sagittal”

Else: video_orientation = “coronal”

7. **Detect the direction of motion**

  If orientation is coronal: trunk_length_diff = abs(euclidean_distance(KP0, KP8)[0] − euclidean_distance(KP0, KP8)[L − 1])

If trunk_length_diff < 0: direction = frontal

Else: direction = rear

  If orientation is sagittal: nose_x_diff = (KP0)[0][0] − (KP0)[0][L − 1]

If nose_x_diff < 0: direction = left-to-right

Else: direction = right-to-left

8. **Identify the four distinct gait events**

  Determine foot strike and foot off events using the Zeni et al. method [33]

  left_foot_strike_raw = euclidean_distance (KP21[0], KP8[0])

  left_foot_off_raw = euclidean_distance ((KP19[0] + KP20[0])/2, KP8[0])

  right_foot_strike_raw = euclidean_distance (KP24[0], KP8[0])

  right_foot_off_raw = euclidean_distance ((KP22[0] + KP23[0])/2, KP8[0])

  left_foot_strike = search_for_peaks (left_foot_strike_raw, flip = TRUE if direction = right-to-left)

  right_foot_strike = search_for_peaks (right_foot_strike_raw, flip = TRUE if direction = right-to-left)

  left_foot_off = search_for_peaks (left_foot_off_raw, flip = FALSE if direction = right-to-left)

  right_foot_off = search_for_peaks (right_foot_off_raw, flip = FALSE if direction = right-to-left)

  Determine mid-midstance and mid-midswing events using the distance between the toes

toe_distances = sqrt(square(KP19_x − KP22_x) + square(KP19_y − KP22_y))

     mid_midswing = toe_distances.idxmin ()

     mid_midstance = toe_distances.idxmax ()

9. **Allocate gait events to specific strides (strides)**

  right_first = left_foot_strike [0] > right_foot_strike [0]

  if right_first: start = right_foot_strike, other_foot_strike = left_foot_strike

  else: starts = left_foot_strike, other_foot_strike = right_foot_strike

  if len(starts) >= 2:

    for i in range of length(start) − 1): current_foot_strike = start[i], next_foot_strike = start [i + 1], end = next_foot_strike − 1

possible_right_foot_off = right_foot_off [current_foot_strike < right_foot_off < next_foot_strike]

possible_other_foot_strike = other_foot_strike[current_foot_strike < other_foot_strike< next_foot_strike]

possible_left_foot_off = left_foot_off [current_foot_strike < left_foot_off < next_foot_strike]

`stride_midstance = midstances [current_foot_strike < midstances < end]

if (length(possible_right_foot_off) >= 1) and (length(possible_other_foot_strike) >= 1) and (length(possible_left_foot_off) >= 1) and (length(stride_midstance) >= 2):

  ‘Direction’: right-to-left or left-to-right,

  ‘index’: i,

  ‘right_first’: TRUE if right_first else FALSE

  ‘right_foot_strike’: current_foot_strike if right_first else possible_other_foot_strike [0],

  ‘right_foot_off’: possible_right_foot_off [0] if right_first else possible_right_foot_off [0]

  ‘left_foot_strike’: possible_other_foot_strike [0] if right_first else current_foot_strike,

  ‘left_foot_off’: possible_left_foot_off [0] if right_first else possible_left_foot_off [0],

  ‘r_midstance’: stride_midstance [0] if right_first else stride_midstance [1],

  ‘l_midstance’: stride_midstance [1] if right_first else stride_midstance [0],

  ‘start’: current_foot_strike

  ‘end’: next_foot_strike − 1

A. If the video is coronal and frontal view, calculate scores of the following EVGS parameters:

**FUNCTION (#17) MST_Lateral_Trunk_Shift_L (Keypoints):**

Trunk = unit_vector(KP1[row] − KP8[row]), labY = array([0, 1])

Trunk_L = (arctan2(Trunk [1], Trunk [0]) − arctan2(labY [1], labY [0])) ∗ 180/pi

     FUNCTION trunk_score(x): If x > 20: Return 2, Else if x < 0 OR (10 < x < 20): Return 1, Else if 0 <= x <= 10: Return 0, Else: Return NaN END

For each stride: midstance_frame = stride[‘l_midstance’]

stride_scores.append (trunk_score(Trunk_L[midstance_frame])) END

**FUNCTION (#17) MST_Lateral_Trunk_Shift_R (Keypoints):**

Trunk = unit_vector(KP1[row] − KP8[row]), labY = array([0, 1])

Trunk_R = (arctan2(Trunk [1], Trunk [0]) - arctan2(labY [1], labY [0])) ∗ 180/pi

     FUNCTION trunk_score(x): If x > 20: Return 2, Else if x < 0 OR (10 < x < 20): Return 1, Else if 0 <= x <= 10: Return 0, Else Return NaN END

For each stride: midstance_frame = stride[‘r_midstance’]

stride_scores.append(trunk_score(Trunk_R[midstance_frame])) END

**FUNCTION (#14) MST_Pelvic_Obliquity_L (Keypoints):**

Hip = unit_vector(KP9[row] − KP12[row]), labY = array([0, 1])

hip_L = (arctan2(Hip [1], Hip [0]) − arctan2(labY [1], labY [0])) ∗ 180/pi

     FUNCTION pelvis_score(x): If −1 < x < 6: Return 0, Else if x < −10 or (x > 15): Return 2, Else if (10 <= x <= −1) or (6 <= x <= 15): Return 1, Else: Return NaN, END

For each stride: midstance_frame = stride[‘l_midstance’]

stride_scores.append(pelvis_score(hip _L[midstance_frame])) END.

**FUNCTION (#14) MST_Pelvic_Obliquity_R (Keypoints):**

Hip = unit_vector(KP9[row] − KP12[row]), labY = array([0, 1])

hip _R = (arctan2(Hip [1], Hip [0]) − arctan2(labY [1], labY [0])) ∗ 180/pi

     FUNCTION pelvis_score(x): If −6 < x < 1: Return 0, Else if x > 10 or (x < −15): Return 2, Else if (1 <= x <= 10) or (−15 <= x <= −6): Return 1, Else: Return NaN, END

For each stride: midstance_frame = stride[‘r_midstance’]

stride_scores.append(pelvis_score(hip _R[midstance_frame])) END

**FUNCTION (#8) MST_Knee_Progression_Angle_L (Keypoints):**

M1 = KP13_y − KP14_y/KP13_x − KP14_x, M2 = KP19_y − KP21_y/KP19_x − KP21_x

Angle = arctan(M1 − M2/1+ (M1 ∗ M2)) ∗ (360/2π)

     FUNCTION ankle_score(x): If x < −25: Return 1, Else if (−25 <= x <= 25): Return 0, Else if x > 25: Return 1, Else: Return NaN, END

For each stride: midstance_frame = stride[‘l_midstance’]

stride_scores.append(ankle_score(Angle[midstance_frame])) END

**FUNCTION (#8) MST_Knee_Progression_Angle_R( Keypoints):**

M1 = KP10_y − KP11_y/KP10_x − KP11_x, M2 = KP22_y − KP24_y/KP22_x − KP24_x

Angle = arctan(M1 − M2/1+ (M1 ∗ M2)) ∗ (360/2π)

     FUNCTION ankle_score(x): If x < −25: Return 1, Else if (−25 <= x <= 25): Return 0 Else if x > 25: Return 1, Else: Return NaN END

For each stride: midstance_frame = stride[‘r_midstance’]

stride_scores.append(ankle_score(Angle[midstance_frame])) END

**FUNCTION (#5) MST_Foot_Rotation_L (Keypoints):**

M1 = KP13_y − KP14_y/KP13_x − KP14_x, M2 = KP19_y − KP21_y/KP19_x − KP21_x

Angle = arctan(M1 − M2/1 + (M1 ∗ M2)) ∗ (360/2π)

     FUNCTION ankle_score(x): If x > 45: Return 2, Else if (25 <= x <= 45): Return 1, Else if (−5 <= x <= 25): Return 0, Else if (−30 <= x <= −5): Return 1, Else if x < −30: Return 2, Else: Return NaN, END

For each stride: midstance_frame = stride[‘l_midstance’]

stride_scores.append(ankle_score(Angle[midstance_frame])) END

**FUNCTION (#5) MST_Foot_Rotation_R (Keypoints):**

M1 = KP10_y − KP11_y/KP10_x − KP11_x, M2 = KP22_y − KP24_y/KP22_x − KP24_x

Angle = arctan(M1 − M2/1 + (M1 ∗ M2)) ∗ (360/2π)

     FUNCTION ankle_score(x): If x < −45: Return 2, Else if (−45 <= x <= 25): Return 1, Else if (−25 <= x <= 5): Return 0 Else if (5 <=x <= 30): Return 1, Else if x > 30: Return 2, Else: Return NaN END

For each stride: midstance_frame = stride[‘r_midstance’]

stride_scores.append(ankle_score(Angle[midstance_frame])) END

B. If the video is coronal and rear view, calculate scores of the following EVGS parameters:

**FUNCTION (#4) MST_Hindfoot_Valgus_Varus_L (Keypoints):**

Hindfoot = unit_vector(KP11[row] − KP24[row]), labY = array([0, 1])

foot_L = (arctan2(Hindfoot [1], Hindfoot [0]) − arctan2(labY [1], labY [0])) ∗ 180/pi

     FUNCTION foot_score(x): If x < −15: Return 2, Else if (−15 <= x <= −6): Return 1, Else if (−6 <= x <= 1): Return 0 Else if (1 <= x <= 10): Return 1, Else if (x >10): Return 2, Else: Return NaN, END

For each stride: midstance_frame = stride[‘l_midstance’]

stride_scores.append(foot_score(foot_L[midstance_frame])) END

**FUNCTION (#4) MST_Hindfoot_Valgus_Varus_R (Keypoints):**

Hindfoot = unit_vector(KP11[row] − KP24[row]), labY = array([0, 1])

foot_R = (arctan2(Hindfoot [1], Hindfoot [0]) − arctan2(labY [1], labY [0])) ∗ 180/pi

     FUNCTION foot_score(x): If x < −15: Return 2, Else if (−15 <= x <= −6): Return 1, Else if (−6 <= x <= 1): Return 0 Else if (1 <= x <= 10): Return 1, Else if (x >10): Return 2, Else: Return NaN END.

For each stride: midstance_frame = stride[‘r_midstance’]

stride_scores.append(foot_score(foot_R[midstance_frame])) END

C. If the video is Sagittal, calculate scores of the following EVGS parameters:

**FUNCTION (#13) IC_Peak_Hip_Flexion (Keypoints):**

M = KP1_y − KP8_y/KP1_x − KP8_x, M1 = 1/M If left-to-right: M2 = KP9_y − KP10_y/KP9_x − KP10_x Else:

M2 = KP12_y − KP13_y/KP12_x − KP13_x

Angle = arctan(M1 − M2/1 + (M1 ∗ M2)) ∗ (360/2π)

     FUNCTION hip_score(x): If (x > 60): Return 2 Else if (45 < x <= 60): Return 1 Else if (25 < x <= 45): Return 0, Else if (10 < x <= 25): Return 1 Else if (x <= 10): Return 2 Else: Return NaN END

For each stride: heelstrike_frame = stride[‘right_foot_strike’] if left-to-right else stride[‘left_foot_strike’]

stride_scores.append(hip_score(Angle[heelstrike_frame])) END

**FUNCTION (#10) IC_Knee_Extension (Keypoints):**

If left-to-right: M1 = KP9_y − KP10_y/KP9_x − KP10_x, M2 = KP10_y − KP11_y/KP10_x − KP11_x

Else: M1 = KP12_y − KP13_y/KP12_x − KP13_x, M2 = KP13_y − KP14_y/KP13_x − KP14_x

Angle = arctan(M1 − M2/1 + (M1 ∗ M2)) ∗ (360/2π)

     FUNCTION knee_score(x): If (x > 30): Return 2, Else if (15 < x <= 30): Return 1, Else if (5 < x <= 15): Return 0, Else if (−10 < x <= 5): Return 1, Else if (x < −10): Return 2 Else: Return NaN END

For each stride: heelstrike_frame = stride[‘right_foot_strike’] if left-to-right else stride[‘left_foot_strike’]

stride_scores.append(knee_score(Angle[heelstrike_frame])) END

**FUNCTION (#1) IC_Initial_Contact (Keypoints):**

If left-to-right: foot = unit_vector(KP22[row] − KP24[row] Else: foot = unit_vector(KP19[row] − KP21[row]

labY = array([1, 0]) Angle = (arctan2(foot [1], foot [0]) − arctan2(labY [1], labY [0])) ∗ 180/pi

     FUNCTION foot_score(x): If (x < 0): Return 2 Else if (0<= x <= 20) or (160<= x <= 180): Return 1 Else: Return 0 END

For each stride: heelstrike_frame = stride[‘right_foot_strike’] if left-to-right else stride[‘left_foot_strike’]

stride_scores.append(foot_score(Angle[heelstrike_frame])) END

**FUNCTION (#16) MST_Peak_Sagittal_Trunk (Keypoints):**

Trunk = unit_vector(KP1[row] − KP8[row]), labY = array([0, 1])

Angle = (arctan2(Trunk [1], Trunk [0]) − arctan2(labY [1], labY [0])) ∗ 180/pi

     FUNCTION trunk_score(x): If x > 15: Return 2, Else if x < −5 OR (5 < x < 15): Return 1, Else if −5 <= x <= 5: Return 0 Else Return NaN END

For each stride: midstance_frame = stride[‘r_midstance’] if left-to-right else stride[‘l_midstance’]

stride_scores.append(trunk_score(Angle[midstance_frame])) END

**FUNCTION (#15) MST_Pelvic_Rotation (Keypoints):**

If left-to-right: Pelvis = unit_vector(KP9[row] − KP12[row] Else: Pelvis = unit_vector(KP12[row] − KP9[row]

labY = array([0, 1]) Angle = (arctan2(Pelvis [1], Pelvis [0]) − arctan2(labY [1], labY [0])) ∗ 180/pi

     FUNCTION pelvic_score(x): If (x < −15) or (x > 21): Return 2 Else if (11 < x < 21) or (−15 < x < −6): Return 1 Else if −6 < x < 11: Return 0 Else Return NaN END

For each stride: midstance_frame = stride[‘r_midstance’] if left-to-right else stride[‘l_midstance’]

stride_scores.append(pelvic_score(Angle[midstance_frame])) END

**FUNCTION (#2) MST_Heel_Lift (Keypoints):**

If left-to-right: foot = unit_vector(KP22[row] − KP24[row] Else: foot = unit_vector(KP19[row] − KP21[row]

labY = array([1, 0]) Angle = (arctan2(foot [1], foot [0]) − arctan2(labY [1], labY [0])) ∗ 180/pi

     FUNCTION lift_score(p, l, Cc,a): If (−10 <= a <= 5) or (−180 <= a <= −170) or ((170 <= a <= 180)): If l < p <Cc: Return 0

     Else if p >= Cc: Return 1 Else if p <= l: Return 1 Else: Return 2 END

For each stride: p = stride[‘right_foot_off] if left-to-right else stride[‘left_foot_off] l= stride[‘r_midstance’] if left-to-right else stride[‘l_midstance’] Cc = stride[‘right_foot_strike] if left-to-right else stride[‘left_foot_strike] m = stride[‘r_midstance’] if left-to-right else stride[‘l_midstance’] stride_scores.append(lift_score(p,l,Cc,Angle[m])) END

**FUNCTION (#12) TST_Peak_Hip_Extension (Keypoints):**

M = KP1_y − KP8_y/KP1_x − KP8_x , M1 = 1/M, left-to-right: M2 = KP9_y − KP10_y/KP9_x − KP10_x Else:

M2 = KP12_y − KP13_y/KP12_x − KP13_x

Angle = arctan(M1 − M2/1 + (M1 ∗ M2)) ∗ (360/2π)

     FUNCTION hip_score(x): If (x > 15): Return 2 Else if (0 < x <= 15): Return 1 Else if (−20 < x <= 0): Return 0 Else if (−35 < x <= −20): Return 1 Else if (x <= −35): Return 2 Return NaN END

For each stride: heelstrike_frame = stride[‘left_foot_strike’] if left-to-right else stride[‘right_foot_strike’]

stride_scores.append(hip_score(Angle[heelstrike_frame])) END

**FUNCTION (#9) TST_Knee_Extension (Keypoints):**

If left-to-right: M1 = KP9_y − KP10_y/KP9_x − KP10_x M2 = KP10_y − KP11_y/KP10_x − KP11_x

Else: M1 = KP12_y − KP13_y/KP12_x − KP13_x. M2 = KP13_y − KP14_y/KP13_x − KP14_x

Angle = arctan(M1 − M2/1 + (M1 ∗ M2)) ∗ (360/2π)

     FUNCTION knee_score(x): If (x > 25): Return 2 Else if (15 < x <= 25): Return 1 Else if (0 < x <= 15): Return 0 Else if (−10 < x <= 0): Return 1 Else if (x <= −10): Return 2 Else: Return NaN END

For each stride: heelstrike_frame = stride[‘left_foot_strike’] if left-to-right else stride[‘right_foot_strike’]

stride_scores.append(knee_score(Angle[heelstrike_frame])) END

**FUNCTION (#3) TST_Ankle_Dorsiflexion (Keypoints):**

If left-to-right: M1 = KP10_y − KP11_y/KP10_x − KP11_x M2 = KP22_y − KP24_y/KP22_x − KP24_x

Else: M1 = KP13_y − KP14_y/KP13_x − KP14_x1 M2 = KP 9_y − KP21_y/KP19_x − KP21_x

Angle = arctan(M1 − M2/1 + (M1 ∗ M2)) ∗ (360/2π)

     FUNCTION ankle_score(x): If (x > 40): Return 2 Else if (25 < x <= 40): Return 1 Else if (5 < x <= 25): Return 0 Else if (−10 < x <= 5): Return 1 Else if (x <= −10): Return 2 Else: Return NaN END

For each stride: heelstrike_frame = stride[‘left_foot_strike’] if left-to-right else stride[‘right_foot_strike’]

stride_scores.append(ankle_score(Angle[heelstrike_frame])) END

**FUNCTION (#11) MSW_Knee_Flexion (Keypoints):**

If left-to-right: M1 = KP9_y − KP10_y/KP9_x − KP10_x M2 = KP10_y − KP11_y/KP10_x − KP11_x

Else: M1 = KP12_y − KP13_y/KP12_x − KP13_x M2 = KP13_y − KP14_y/KP13_x − KP14_x

Angle = arctan(M1 − M2/1 + (M1 ∗ M2)) ∗ (360/2π)

     FUNCTION knee_score(x): If (x > 85): Return 2 Else if (70 < x <= 85): Return 1 Else if (50 < x <= 70): Return 0 Else if (35 < x <= 50): Return 1 Else if (x <= 35): Return 2 Else: Return NaN END

For each stride: heelstrike_frame = stride[‘l_midstance] if left-to-right else stride[‘r_midstance’], stride_scores.append(knee_score(Angle[heelstrike_frame])) END

**FUNCTION (#7) MSW_Ankle_Dorsiflexion (Keypoints):**

If left-to-right: M1 = KP10_y − KP11_y/KP10_x − KP11_x M2 = KP22_y − KP24_y/KP22_x − KP24_x

Else: M1 = KP13_y − KP14_y/KP13_x − KP14_x M2 = KP19_y − KP21_y/KP19_x − KP21_x

Angle = arctan(M1 − M2/1 + (M1 ∗ M2)) ∗ (360/2π)

     FUNCTION ankle_score(x): If (x > 30): Return 2 Else if (15 < x <= 30): Return 1 Else if (−5 < x <= 15): Return 0 Else if (−20 < x <= −5): Return 1 Else if (x <= −20): Return 2 Else: Return NaN END

For each stride: heelstrike_frame = stride[l_midstance] if left-to-right else stride[‘r_midstance’]

stride_scores.append(ankle_score(Angle[heelstrike_frame])) END

**FUNCTION (#6)MSW_Foot_Clearance(Keypoints):**

fh = euclidean_distance(KP24[0] if left-to-right else KP21[0], KP8[0])

sh = euclidean_distance(KP21[0] if left-to-right else KP24[0], KP8[0])

ft = euclidean_distance(KP22[0] if left-to-right else KP19[0], KP8[0])

st = euclidean_distance(KP19[0] if left-to-right else KP22[0], KP8[0])

ml = euclidean_distance((KP10[0] if left-to-right else KP13[0] + KP24[0] if left-to-right else KP21[0])/2, KP8[0])

     FUNCTION foot_score(ft,st,ml,fh,sh): If ( ml >= ft): Return 1 Else if ((st >= ft) and ( sh >= fh)): Return 0 Else if ((sh < fh) and (st < ft)): Return 2 Else if ((st < ft) and (sh > fh)): Return 1 Else: Return NaN END

For each stride: heelstrike_frame = stride[l_midstance] if left-to-right else stride[‘r_midstance’]

stride_scores.append(ankle_score(ft[heelstrike_frame],st[heelstrike_frame],ml[heelstrike_frame], fh[heelstrike_frame], sh[heelstrike_frame])) END
